# Design and Characterization of a Cell-Penetrating Peptide Derived from the SOX2 Transcription Factor

**DOI:** 10.3390/ijms22179354

**Published:** 2021-08-28

**Authors:** Neha S. Gandhi, Edina Wang, Anabel Sorolla, Yu Jie Kan, Adil Malik, Jyotsna Batra, Kimberly A. Young, Wan Jun Tie, Pilar Blancafort, Ricardo L. Mancera

**Affiliations:** 1 Curtin Medical School, Curtin Health Innovation Research Institute and Curtin Institute for Computation, Curtin University, GPO Box U1987, Perth, WA 6845, Australia; neha.gandhi@qut.edu.au (N.S.G.); yujiekan@gmail.com (Y.J.K.); kimberly.young@postgrad.curtin.edu.au (K.A.Y.); 2 Centre for Genomics and Personalised Health, School of Chemistry and Physics, Queensland University of Technology, 2 George Street, Brisbane, QLD 4000, Australia; 3 Cancer Epigenetics Group, Harry Perkins Institute of Medical Research, School of Anatomy, Physiology and Human Biology, The University of Western Australia, 35 Stirling Highway, Crawley, WA 6009, Australia; edina.wang@perkins.org.au (E.W.); asorolla@irblleida.cat (A.S.); ash.tie@uwa.edu.au (W.J.T.); 4 Lleida Institute for Medical Research Dr Pifarré Foundation, 25198 Lleida, Spain; 5 School of Biomedical Sciences, Queensland University of Technology, Brisbane, QLD 4059, Australia; m.malik@hdr.qut.edu.au (A.M.); jyotsna.batra@qut.edu.au (J.B.); 6 Australian Prostate Cancer Research Centre-Queensland, Translational Research Institute, Woolloongabba, QLD 4102, Australia; 7 The Greehey Children’s Cancer Research Institute, The University of Texas Health Science at San Antonio, San Antonio, TX 78229, USA

**Keywords:** iPep, cell-penetrating peptides, SOX2, homeodomain, molecular dynamics, cellular internalization

## Abstract

SOX2 is an oncogenic transcription factor overexpressed in nearly half of the basal-like triple-negative breast cancers associated with very poor outcomes. Targeting and inhibiting SOX2 is clinically relevant as high SOX2 mRNA levels are positively correlated with decreased overall survival and progression-free survival in patients affected with breast cancer. Given its key role as a master regulator of cell proliferation, SOX2 represents an important scaffold for the engineering of dominant-negative synthetic DNA-binding domains (DBDs) that act by blocking or interfering with the oncogenic activity of the endogenous transcription factor in cancer cells. We have synthesized an interference peptide (iPep) encompassing a truncated 24 amino acid long C-terminus of SOX2 containing a potential SOX-specific nuclear localization sequence, and the determinants of the binding of SOX2 to the DNA and to its transcription factor binding partners. We found that the resulting peptide (SOX2-iPep) possessed intrinsic cell penetration and promising nuclear localization into breast cancer cells, and decreased cellular proliferation of SOX2 overexpressing cell lines. The novel SOX2-iPep was found to exhibit a random coil conformation predominantly in solution. Molecular dynamics simulations were used to characterize the interactions of both the SOX2 transcription factor and the SOX2-iPep with FGF4-enhancer DNA in the presence of the POU domain of the partner transcription factor OCT4. Predictions of the free energy of binding revealed that the iPep largely retained the binding affinity for DNA of parental SOX2. This work will enable the future engineering of novel dominant interference peptides to transport different therapeutic cargo molecules such as anti-cancer drugs into cells.

## 1. Introduction

Transcription factors (TFs) are proteins capable of binding onto specific DNA sequences and affect the regulation of subsequent transcription by interacting either through their DNA-binding domains (DBDs) or with other TFs and chromatin cofactors and modifiers [1]. Thus, these molecules can be distinguished from other transcriptional regulatory proteins due to their ability to interact with DNA in a sequence-specific manner [2,3].

Particular families of TFs, such as Homeobox genes (HOX genes), which play an important role during embryogenesis and development, are also found differentially expressed in particular subtypes of breast cancers and in other malignancies [4,5,6]. Notably, the SOX (SRY-related High Mobility Group (HMG)-box) family of proteins are related to the SRY (Sex determining Region Y; a sex-determining gene on the Y chromosome in both marsupial and placental mammals) and comprise nearly half of the known HMG-box proteins. These TFs play important roles in a variety of developmental processes, particularly in organogenesis [7,8]. The HMG TF SOX2 is highly expressed in embryonic stem cells (ESCs), in neural progenitor cells [9] and is one of the TFs necessary for the formation of induced pluripotent stem cells from somatic cells [10].

Overexpression of SOX2 has been associated with copy number amplification and promoter overexpression in multiple malignancies such as breast carcinomas, lung cancer tissues including squamous cell carcinoma and adenocarcinomas, with high levels of SOX2 expression correlating with decreased patient survival [11]. Notably, SOX2 deregulation has been demonstrated in 43% of basal-like breast carcinomas, which are triple-negative malignancies lacking the hormonal receptors and HER2. These tumors are originated from breast progenitor cells and consequently are poorly differentiated, aggressive, and associated with a high risk of chemoresistance and relapse. The ectopic overexpression of SOX2 enhances malignant processes such as cell proliferation, migration, and anchorage-independent growth and induces tumorigenesis in vivo [12].

The SOX protein members bind to DNA utilizing a single sequence-specific HMG-box with a similar binding motif of A/TAACAA/T [8]. However, different SOX proteins have distinct preferences for the two nucleotides flanking the AACAAT motif [8]. Importantly, however, the HMG-box of SOX proteins interacts with DNA and engages in numerous protein–protein interactions that are typically mediated by highly divergent non-HMG-box sequences [13]. SOX2 proteins are thus able to accommodate a broad range of sequence-specific DNA bend angles that facilitate the binding of other DBDs to adjacent binding sites [14].

**Figure 1:**
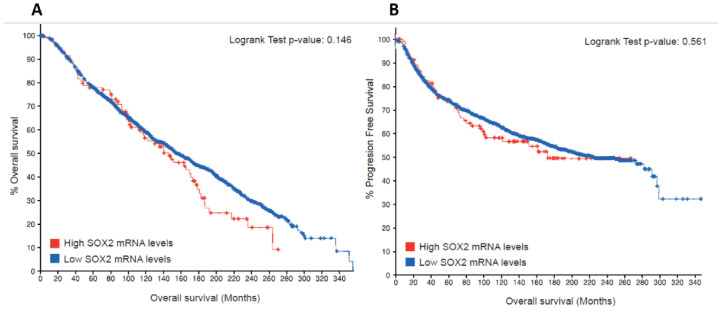
Kaplan-Meier estimated overall survival (**A**) and progression-free survival (**B**) in patients (*n* = 1904) affected by breast cancer from cBioPortal. Patients with low SOX2 mRNA levels (*n* = 106) showed an increased survival rate compared to high SOX2 expressing patients (*n* = 1798).

The X-ray crystal structure of the SOX2/OCT4/FGF4-enhancer complex (PDB structure 1GT0) reveals the nature of the interactions of the HMG domain of SOX2 with DNA and the POU (Pit-Oct-Unc) domain of OCT4 (octamer-binding transcription factor 4) [15,16,17]. OCT4 is a homeodomain TF that recognizes the sequence ATTTGCAT. The SOX2 TF sequence is highly conserved across species, and most of its conserved residues are found to play an important role in the ordering of the C-terminal region of its HMG domain, which includes Val3, Arg5, Pro6, His63, Pro68, Tyr70, Tyr72, Arg75, and Arg76 (Figure 1) [15]. These protein residues are responsible for protein–protein interactions with OCT4 and are highly conserved across the SOX family [15]. The conserved C-terminal loop of SOX2 consists of basic residues that serve as an intrinsic cell- and nuclear-penetrating motif. The FGF4 enhancer is a DNA regulatory element that contains the above-mentioned octamer motif for POU binding and an adjacent motif for HMG binding. In this structure SOX2 binds to the minor groove of the FGF4-enhancer and forms an HMG/DNA interaction surface that is similar in size to that of the POU/DNA interaction. The HMG domain also strongly bends the DNA fragment at approximately 90° towards the major groove. Side chains from residues Met7, Phe10, and Met11 of helix 1 of the HMG domain of SOX2 are inserted between three base pair stacks of the recognition sequence (C^T^^TGTT), which leads to the unwinding of the DNA double strand at the SOX2 binding site [14].

The highly conserved residues of the C-terminal region of the HMG box can be exploited for the design of synthetic interfering peptides (iPeps) that may interfere with the function of SOX2 in breast cancer cells by competitively disrupting the cooperative interactions in the SOX2/OCT4/DNA complex. The use of peptides to interfere with the interactions of proteins involved in cancer has been demonstrated successfully with iPeps comprising a conserved hexamotif and the N-terminus flanking sequence of the homeobox of the transcription factor Engrailed 1 (EN1) in basal-like breast cancer [18,19]. Interestingly, low dose EN1-iPeps can also sensitize breast cancer cells to cancer drugs such as taxol, 5-fluorouracil, or Docetaxel [20]. Bi-functional nanoparticles encapsulating EN1-iPeps and docetaxel selectively decreased the proliferation of basal-like breast cancer cells and inhibited tumor development in a syngeneic model of breast cancer in mice [20,21]. Similar to EN1, we and others have described iPeps selectively inhibiting oncogenic TFs such as c-MYC in breast carcinoma models [21]. 

The customized development of iPeps derived from oncogenic TFs that are overexpressed in aggressive and chemoresistant carcinomas, such as SOX2, can selectively target specific tumors overexpressing these TFs thereby sensitizing these chemoresistant carcinomas to chemotherapy drugs. To the best of our knowledge, these peptides have never been described in the context of SOX family members. 

Herein we describe the development and molecular characterization of a SOX2-derived iPep comprised of the truncated 24 amino acid long C-terminus of SOX2 encompassing residues Lys57-Thr80. For effective cell penetration and intracellular delivery, iPeps must be designed with cell penetration sequences, such as the SV40 sequence to transport hydrophobic cargoes, such as the EN1-iPep [18]. We discovered that our SOX2-iPep comprised an intrinsic and cell penetration sequence (_57_KRLRALH_63_) rich in basic amino acids, which mediated potent intracellular delivery and nuclear localization. The mutation of cationic amino acids Lys57, Arg60, Arg75, and Arg76 to alanine abolished the intracellular delivery and biological activity of the peptide. Molecular dynamics (MD) simulations characterized the interactions of the SOX2-derived iPep with OCT4 and FGF4 enhancer DNA and predicted the associated free energies of binding. Single-residue free-energy decomposition analysis identified the key residues that dominate the interaction of the SOX2-derived iPep with DNA and OCT4. In summary, our approach opens the door to personalized cancer treatment by inhibition of SOX2 using iPeps to block protein–protein interfaces between SOX2 and its molecular effectors potentially. The same approach could be applied to inhibit an extended panel of oncogenic TFs that are hard to drug with current approaches.

## 2. Results and Discussion

### 2.1. Association between SOX2 Expression and Survival of Patients with Breast Cancer

As one rationale for developing cell-penetrating peptides is to target the oncogenic SOX2 TF in breast cancer, we wanted to investigate whether the SOX2 expression levels in breast cancer biopsies were a predictive indicator of overall survival and progression-free survival in these patients. We thus analyzed publicly available datasets from breast cancer patients containing mRNA expression and clinical data. Targeting and inhibiting SOX2 is clinically relevant as high SOX2 mRNA levels positively correlate with decreased overall survival and progression-free survival before 270 months in patients affected with breast cancer (Figure 1).

### 2.2. Peptide Identification and Quantification

**Figure 2:**
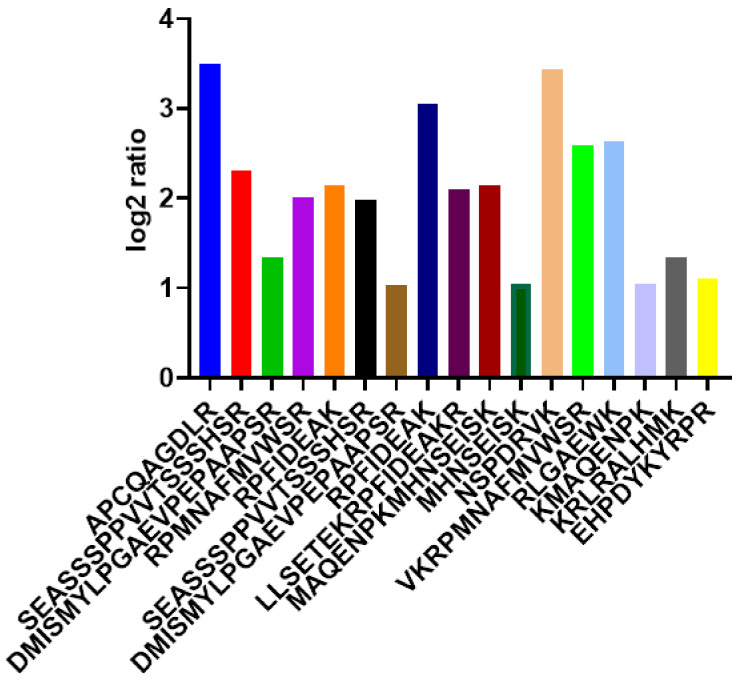
iTRAQ ratios of the tryptic peptides identified and analyzed using Breast cancer Mass Spectrometry data.

For iTRAQ data, proteins having a single peptide were excluded for quantification. The list of identified SOX proteins, the expression ratios and patient information are included in Suppl. Information (excel file). The obtained protein data were checked for peptide levels. For this identification, only multiple peptides (≥2) of SOX proteins identifications were taken into consideration. As represented in Figure 2, peptides coming from SOX proteins were quantified and converted to log2 ratios based on the peptide/total protein ratios. iTRAQ ratios are the intensity of the peptides in the sample/intensity of internal standard. Tryptic peptides, including the highly abundant APCQAGDLR and NSPDRVK, were found in all the patient sample datasets. Homeodomain tryptic peptides including RPMNAFMVWSR, RPFIDEAK, LLSETEKRPFIDEAKR were found to have lower ratios than highly expressed peptides.

### 2.3. Design of a SOX2 Interference Peptide (iPep)

**Figure 3:**
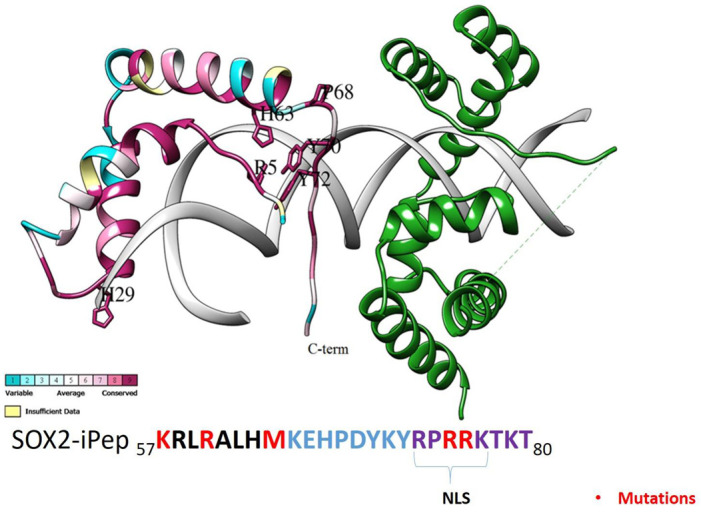
Structure of the HMG domain of SOX2 in complex with the POU domain of OCT4 and the FGF4-enhancer (taken from PDB entry 1GT0). Proteins are shown in their ribbon representation. SOX2 is colored according to evolutionary conservation (Consurf) and OCT4 is shown in green. The FGF4 enhancer is represented as two DNA strands (bases are not shown for clarity). The most highly conserved amino acids in SOX2 are shown in purple sticks. A twenty-four residue long iPep was taken from the conserved SOX2 C-terminus. Residues Arg4 and Arg73-Thr77 are involved in protein–protein contacts with OCT4. Tyr72, Arg75 and Lys77 are directly involved in binding to the minor groove of DNA. Residues Arg73-Lys79 comprises the nuclear localization sequence (NLS). The residues in the SOX2-iPep highlighted in red were mutated to form an alanine-mutated version of the SOX2 iPep.

Figure 1 outlines the SOX2–OCT4 complex with DNA. The SOX2 protein (ConSurf-DB [22]: 1GT0) is shown as a ribbon, colored according to the amino acid (aa) sequence conservation from the most conserved (dark magenta) to the most divergent (dark cyan) based on an alignment of 150 SOX2 sequences from different species. SOX2 proteins harbor two nuclear localization sequences (NLSs), located distally at the N- and C-terminus of the DNA-binding domain. These sequences consist of conserved basic and aromatic hydrophobic residues. The Helix H2 (HMG domain) of SOX2 is also conserved. The most highly conserved amino acids in SOX2 are shown in purple sticks (Figure 3). The conserved residues Arg75 and Arg76 are involved in protein–protein contacts with the binding partner of SOX2, OCT4. In contrast, Tyr72, Arg75, and Lys77 are directly involved in binding to the minor groove of DNA. A twenty-four residue iPep (_57_KRLRALHMKEHPDYKYRPRRKTKT_80_) was derived from the conserved C-terminal domain of SOX2. Importantly, two conserved aromatic residues, Tyr70 and Tyr72, are present in the SOX2-iPep and located in the core of the sequence. These aromatic residues are required for intramolecular CH–pi and pi–pi interactions, as well as for intermolecular interactions with binding partners as observed in the EN1 and HOX peptides [23]. These conserved aromatic residues are flanked by sequence-rich and positively charged Arg/Lys residues, which can potentially mediate electrostatic interactions with the negatively charged plasma membrane. We chose this specific sequence from C-terminal SOX2 as it has been reported to be effective in mediating the penetration of peptide cargos containing hydrophobic residues, such as W and Y [24].

Although there are no naturally occurring mutants in the NLSs of SOX2 that have been documented, sex-determining region Y (SRY) mutants R62G, R75M, and R76P have been shown to impede nuclear localization and result in sex reversal. As a control, we generated a SOX2-iPep mutant in which the cationic residues Lys57, Arg60, Arg75, and Arg76, and one norleucine (Nle64) were mutated to alanine. These mutations were expected to abolish the activity of the SOX2-iPep. This mutant SOX2-iPep was used to control the toxic effects unrelated to SOX2-regulated genes. This peptide variant is identical to the active SOX2-iPep except for the specific amino acids necessary for the interaction of SOX2 with its binding partners and the DNA, ultimately blocking SOX2-dependent oncogenic transcription.

### 2.4. Cellular Internalization and Biological Effects of SOX2 iPep in Basal-like Breast Cancer Cells

**Figure 4:**
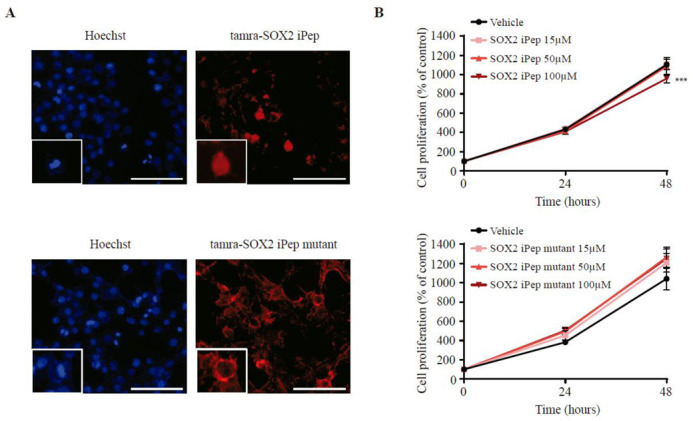
(**A**) Representative fluorescence images of T11 cells treated with 20 µM of SOX2 iPep conjugated with TAMRA (TAMRA-CKRLRALH-Nle-KEHPDYKYRPRRKTKT-NH_2_), and SOX2 iPep mutant conjugated with TAMRA for 2 h. Bars represent 100 µm. (**B**) Cell proliferation plot of T11 cells treated with 15 µM, 50 µM, and 100 µM of SOX2 iPep and SOX2 iPep mutant for 24 and 48 h. Treatment conditions were compared statistically to the vehicle control using a two-way ANOVA test. ns means not significant, *** means *p* < 0.0005.

In order to investigate cellular internalization and biological effects of the parental SOX2-iPep (TAMRA-CKRLRALH-Nle-KEHPDYKYRPRRKTKT-NH_2_) in basal-like breast cancer, we performed immunofluorescence (IF) and cell proliferation assays, respectively. We synthesized a parental and mutant SOX2-iPep N-terminally conjugated with TAMRA, respectively, which facilitated the intracellular detection of the peptide by IF (Figure 4A). A similar approach has been used for the characterization of an OCT4 peptide with a protein transduction domain capable of translocating into human and mouse cells [25]. We found that the SOX2-iPep was internalized very rapidly and effectively in 4.9% of T11 cells within two hours and co-localized with Hoechst staining into the nucleus of the breast cancer cells (Supplementary Figure S1). Interestingly, the SOX2-iPep was precisely localized in specific chromatin foci, as indicated by the dotted pattern of the staining. In contrast, treatment with the alanine-mutated form of the SOX2-iPep resulted primarily in plasma membrane-associated (with minor cytoplasmic) staining indicating that this peptide was almost unable to enter the nucleus, only in 0.44% of them concretely (*p* = 0.000702). Its localization was mainly cytoplasmatic, 74.8% of cells presented such staining while for the SOX2-iPep it was 38.2% (*p* = 0.0081). These findings suggest that the SOX2-iPep localizes into the nuclei of the tumor cells via an intrinsic “RPRRKTKT” cell penetration/nuclear localization sequence.

We next investigated the capacity of the SOX2-iPeps to inhibit the growth of the triple-negative breast cancer cell line T11. The importance of SOX2 in maintaining stem cell state in embryonic stem cells has been previously described [10]. Additionally, SOX2 inhibition by shRNA reduced cell proliferation in adult stem cells [26] and glioblastoma cells [27]. Moreover, our group demonstrated proliferation inhibition in basal-like breast cancer cell lines after targeting SOX2 with zinc-finger (ZF)-based artificial transcription factors both in vitro and in vivo [28]. Similarly, we have observed a modest but significant reduction (*p* = 0.0001) in cell proliferation of 13.16% after treatment of T11 cells with the SOX2-iPep (Figure 4B). We also tested both the SOX2-iPep and mutant SOX2-iPep on two SOX2-enriched cell lines, MCF-7 and PA-1, and found that when the cells were treated with the active form of SOX-iPep, cell growth was reduced by 9.5% and 26.4%, respectively (Supplementary Figure S2A,B), and it was significant at 100 µM. However, the human dermal epithelial fibroblasts (HDEF) cells were less affected by the SOX-iPep treatment (Supplementary Figure S2C). Interestingly, We found that the mutant SOX2 iPep also elicited a significant decrease in cell proliferation in the PA-1 cell lines (17.8%). Because this mutant peptide is not localized in the nucleus, the activity of the mutant peptide could be explained by interference with membrane-associated signaling similar to that which we have observed with other membrane-associated peptides, such as melittin [29].

The anti-cancer activity of the SOX2-iPep was confirmed by the assessment of cell death assays. We found that the active SOX2-iPep significantly induced 27.6% and 13.7% of apoptotic cell death in both MCF7 and PA-1 cells, respectively, as observed by a TUNEL assay (Supplementary Figure S3). The lower percentages of cell proliferation compared to the percentages of apoptotic cells can be explained by the presence of very early apoptotic cells. These cells are still metabolically active and still capable of reducing the MTT, but they show nuclei pyknosis with Hoechst staining and thus are recorded as apoptotic cells. Another possibility is that the SOX2-iPep could induce metabolic hyperactivation in MCF7 and PA-1 cells whilst cells already reflect apoptotic features. Similarly, Rai et al. reported a discrepancy between the decrease in MTT reduction and the reduction of cell numbers when various cell lines were subjected to radiation [30]. The authors attributed such disagreement to a mitochondrial metabolic hyperactivation induced by radiation [30].

It has been described that cancer with stem-cell-like characteristics presents high levels of SOX2. This is occurring in basal-like breast cancers where SOX2 has been found to be preferentially expressed in this breast cancer subtype and identified as a driver of their poorly differentiated phenotype [31]. Similarly, mammospheres derived from MCF7 cells show higher levels of SOX2 expression compared with the parental cell line [32]. Regarding the new cell lines analyzed, MCF7 and PA-1 cells express similar levels of SOX2 [33], which correlates with similar sensitivity of the cells to the SOX2-iPep. T11 cells overexpress SOX2 in comparison with the mice embryonic fibroblasts NIH/3T3, a suitable murine cell control (Supplementary Figure S4)

Regarding the translation of the SOX2 iPep, it is important to perform further engineering of the peptide to minimize its action in stem and progenitor cells. One plausible strategy could be the linkage of RGD peptides which have a high affinity for αvβ3 and αvβ6 integrins present in high abundance on the breast cancer surface, as previously described by our group [21]. Such RGD linkage will confer targeting selectivity to the SOX2-iPep for breast cancer cells while avoiding undesirable effects in stem and progenitor cells. This engineering step could be the basis of further investigations.

### 2.5. Secondary Structure of SOX2 iPEP

**Figure 5:**
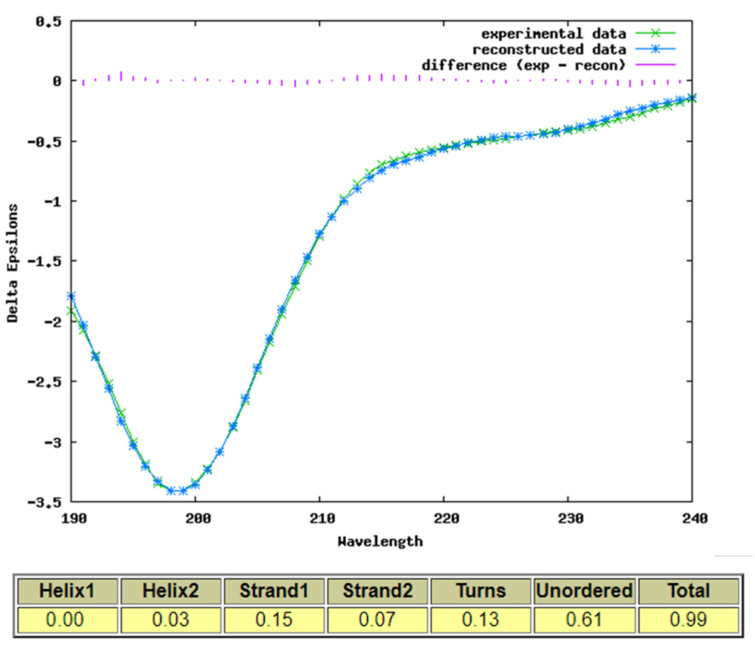
Circular dichroism of the Sox-2 iPep: CKRLRALH-Nle-KEHPDYKYRPRRKTKT. CD spectrum of the Sox-2 iPep at 50 µM concentrations in 50 mM PB, pH 7.4 at 4C reveals that there is no helical content and, instead of that, the peptide has beta-strand content (~22%), and the rest is a random coil.

We used direct experimental evidence from CD measurements to investigate the secondary structure content of the iPep (FITC-CKRLRALH-Nle-KEHPDYKYRPRRKTKT-NH_2_) in solution in the absence of DNA (Figure 5). The resulting CD spectrum shows that the SOX2 peptide is predominantly unstructured in solution.

The structure of the FITC-labelled SOX2 iPep is different from the NMR structure of full-length SOX2 in solution and in the complex with DNA and OCT4. The region ^57^KRLRALHMKEH^67^ is helical in the NMR and X-ray structures of SOX2. The region has positively charged residues (R and K) and Ala and Leu residues that should have, in principle, a strong tendency to exhibit helical conformation. However, it is important to note that the non-polar residues in the helical portion that would be part of the iPep (Ala and Leu) are part of a cluster of hydrophobic interactions with another helix and coil in the N-terminal region of the HMG box in full-length SOX2. In addition, His63 (which the NMR structure shows is protonated at the experimental pH of 6.7) [34] in the iPep sequence also forms an H-bond with the N-terminal coil region in the HMG box. This suggests that in the absence of the rest of the sequence in the HMG box, the presumed helical segment of the iPep is no longer stabilized in its helical conformation due to the loss of the above interactions with the neighboring helix and coil. A recent structure of SOX2 NLS [35] from the N- and C-terminal (similar in sequence to SOX2 iPep) bound to importin-α in the minor groove and major groove also showed disordered conformation in alignment with the CD data. In contrast, when bound to nucleosomes [36,37], these NLS regions are in close proximity, and in a closed conformation in particular the region ^57^KRLRALHMKEH^67^ is helical.

A number of peptides as well as proteins from the large family of transcriptional regulators such as Engrailed-2, Hoxa5, Hoxc8, PDX-1, Pax-4, FITC-labelled penetratin, and OCT4, have been assessed for their capacity to cross cellular membranes into the cytoplasm and/or nuclei. The relative importance of the secondary structure of these peptides for uptake is to date not fully understood [38]. When bound to lipid membranes, both FITC-labelled OCT4 peptide and penetratin were found to adopt α-helical conformations, and the unlabeled OCT4 peptide exhibited a disordered structure when free in solution or bound to lipid membranes. The unlabeled and labeled OCT4 and penetratin not only internalized but were shown to translocate entire proteins such as Cre and its natural cargo [25,39]. Owing to the role of SOX2 in triple-negative breast cancer and its inherent cell-penetrating sequence, this SOX2 iPep when physically linked with small molecules such as doxorubicin or cisplatin (cargo) might help to localize them specifically in the nucleus of cancer cells overexpressing SOX2. Similar approaches such as fusing the internalization sequence of Antennapedia to a 14 residue peptide from Myc [40] and combination therapy of docetaxel and Myc peptide [41] have been used for nuclear-targeted anti-cancer drug delivery.

### 2.6. MD Simulations

Suppl Figure S5 shows the time evolution of the root mean square deviation (RMSD) of the main chain of SOX2/OCT4 and iPep/OCT4 averaged over the ten independent MD simulations of each corresponding system. The RMSD values in the SOX2/OCT4/FGF4-enhancer system were found to oscillate between 2.0 and 2.5 Å, whereas the RMSD values of the iPep/OCT4/FGF4-enhancer system were found to oscillate between 2.5 Å and 3.0 Å, with respect to the crystal structure. This suggests that there is no significant change in the conformation of the proteins and peptides in both systems. The somewhat higher RMSD values of the main chain in the iPep/OCT4 structure are within expectation as the iPep is a C-terminal truncation of SOX2 and has fewer interactions with DNA compared to the full-length HMG domain of SOX2. The iPep nonetheless maintains a stable, DNA-bound conformation.

The time evolution of the Root Mean Square Deviation (RMSD) of the main chain of SOX2 and the DNA backbone in the absence of OCT4 is shown in Figure S6, corresponding to the average over ten independent MD simulations. The RMSD values in the SOX2 C-terminus were found to oscillate between 4.0 and 5.0 Å, whereas the RMSD values of the DNA backbone were found to oscillate around 1.5 Å compared to the crystal structure. This suggests that there is a significant change in the conformation of the C-terminal region in the absence of OCT4.

#### 2.6.1. Free Energies of Binding of SOX2 and iPep to DNA in the Presence and Absence of OCT4

The free energies of binding (ΔG_binding_) of SOX2/iPep in both the SOX2/OCT4/FGF4-enhancer and the iPep/OCT4/FGF4-enhancer systems were calculated using the MM/GBSA and MM/PBSA methods and are reported in Table 1. Figure S7 shows the time evolution of the average ΔG_binding_ for SOX2 and the iPep. The ΔG_binding_ of SOX2 and iPep predicted by both the MM/GBSA and MM/PBSA methods oscillate in a consistent range of 10 kcal/mol, reflecting the stability of the complexes throughout the MD simulations.

The average predicted ΔG_binding_ obtained with the MM/GBSA method was −192.7 kcal/mol for SOX2 and −55.8 kcal/mol for the iPep, while the average ΔG_binding_ obtained with the MM/PBSA method was −74.1 kcal/mol for SOX2 and −11.4 kcal/mol for the iPep. While the absolute values of the free energies of binding predicted by implicit solvation methods are not reliable (hence the large differences between the predictions by MM/GBSA and MM/PBSA), their relative values are. The iPep is predicted to have a weaker binding affinity to DNA compared to SOX2, which is expected given the truncated nature of the iPep. The iPep has fewer interactions with DNA compared to SOX2, specifically the interactions between the N-terminus of SOX2 and DNA. On the other hand, the interactions between SOX2 and OCT4 involve the C-terminus of SOX2, which are fully preserved in the iPep. The free energy components predicted by both implicit solvation methods (Table 1) indicate that van der Waals interactions make the largest contribution to binding, reflecting the large interface between protein and DNA. It is interesting to note that the free energy terms arising from the interactions and solvation of electric charges in the macromolecules (ΔE_elec_ + ΔG_GB_, in the case of MM/GBSA, or ΔE_elec_ + ΔG_PB_ in the case of MM/PBSA, as shown in Table 1) reveal that the sum is more favorable (it has lower values) for SOX2 than for the iPep. This is likely due to the presence of specific water molecules at the SOX2/DNA interface (see section below). These water molecules likely reduce the magnitude of the favorable electrostatic interactions upon binding (which is reflected indirectly in the larger distances between charged groups bridged by water molecules at the interface), an effect that appears to have a relatively smaller impact in SOX2 compared to the iPep.

**Figure 6:**
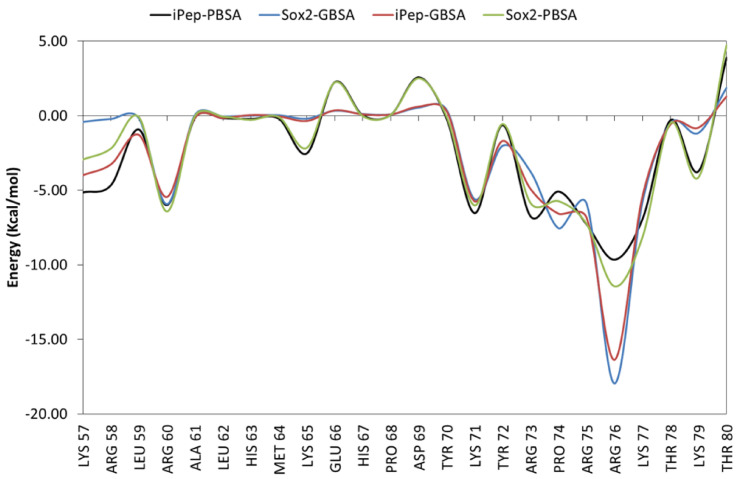
Free energy of binding decomposition per amino acid residue in the iPep and the corresponding C-terminus of SOX2. The free energies were calculated with both the PBSA and GBSA methods. SOX2 numbering is taken into account for this analysis.

To further characterize the binding affinity retained in the iPep compared to SOX2, decomposition was carried out of the free energy of binding (ΔG) to DNA into the contributions of each constituent amino acid of the iPep compared to the corresponding region in the C-terminus of SOX2, as shown in Figure 6. The first two residues of the iPep (corresponding to residues Lys57 and Arg58 in SOX2) are predicted to have a larger favorable contribution to the free energy of binding compared to the same residues in SOX2. This is due to optimizing the electrostatic interactions of these residues with DNA in the iPep (Table S1). The remaining 22 residues in the iPep have similar free energy contributions to those of the corresponding residues in SOX2. The free energy of binding obtained from residue decomposition analyses of all of the residues (Ly57-Thr80) in the iPep is ~−60 kcal/mol and ~−58 kcal/mol, as predicted by the MM/GBSA and MM/PBSA methods, respectively, whereas the total contribution to the free energy of binding of the corresponding residues in SOX2 is ~−53 kcal/mol and −54 kcal/mol, as predicted by the MM/GBSA and MM/PBSA methods, respectively. These figures suggest that the iPep has not only retained but in fact improved the binding affinity to DNA of the corresponding region of SOX2 (Figure 6). It is important to note that the sum of the free energy contributions of all amino acids from the free energy decomposition analysis differs from the total free energy reported in Table 1 because the entropy and internal strain energy are not part of the calculation.

Amino acid residues that contribute ≤−5 kcal/mol to the free energy of binding may be considered as interaction hot spots in SOX2. The region defined by residues Ala61-Tyr70 is helical in structure and does not interact with DNA (Figure 6). Arg60 is part of the local helical structure of the peptide and its side chain forms an electrostatic interaction with the phosphate in DT9 of DNA in the SOX2/OCT4/FGF4-enhancer simulation (Table S1).

**Figure 7:**
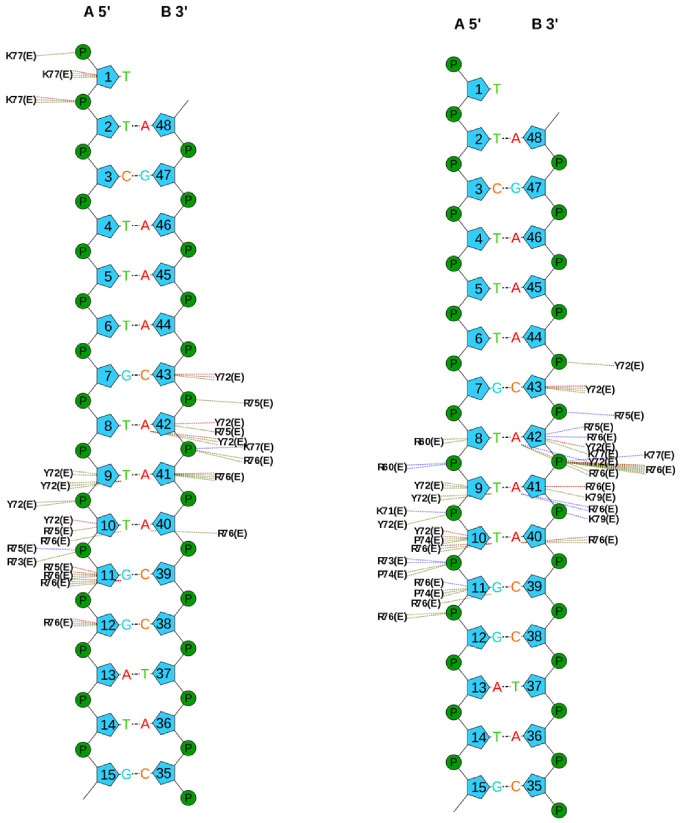
Scheme of the interactions with DNA of residues in SOX2 (chain E) corresponding to the iPep in PDB entry 1GT0 (**left**) and a representative snapshot from the MD simulation of SOX2/OCT4/FGF4-enhancer (**right**). Hydrogen bonds, ionic interactions, and vdW interactions are shown by blue, brown, and green dotted lines. The plots were obtained using the NuProPlot [41]. Bridging water molecules are not shown. Residues Lys57 to Tyr70 are involved in inter-residue interactions in the SOX2 C-terminal helix and do not interact with DNA in the starting structure whereas Arg60 forms an ionic interaction with DNA (DT8 and DT9). Numbering is based on the SOX2 crystal structure.

During the MD simulation, the side chain of Lys71 in both SOX2 and the iPep interacts with the phosphate backbone of DT10, but this interaction is not observed in the crystal structure (Table S1). Tyr72 has unique interactions since its bulky side chain makes a large contribution to vdW interactions while making an overall unfavorable electrostatic interaction (Figure S8 and Figure 7). Tyr72 is required for positioning the C-terminal in the minor grove. The side chain of Arg73 is involved in an ionic interaction with the phosphate in DG11 and Asp29 of OCT4, whereas the backbone amide forms hydrogen bonds with the phosphate in DC43 (Figure S9). The side chains of basic residues Arg75, Lys77, and Lys79 form ionic interactions with the phosphate groups in DC43, DA42, and DA41, respectively (Table S1). The side chain of Arg76 in SOX2 forms hydrogen bonds with the purine rings of DA41 and DG11, and retains the original hydrogen bond with the ribose of DG11 observed in the crystal structure. Both Thr78 and Thr80 form inter-residue hydrogen bonds with other neighboring residues of SOX2. It appears that Arg60, Lys71, and Arg76 are the most important residues that determine the binding affinity of the SOX2 iPep.

**Figure 8:**
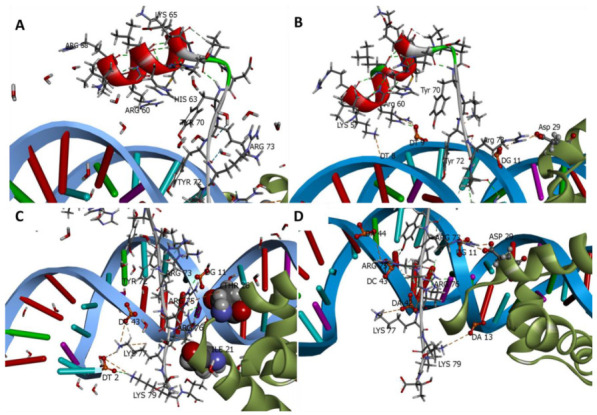
Interactions of residues in SOX2 (chain E) corresponding to the iPep in PDB entry 1GT0. The SOX2 region corresponding to the iPep is shown as ribbon and colored according to secondary structure. The FGF4-enhancer (DNA) and OCT4 are shown in blue and green ribbon representation, respectively. Hydrogen bonds, ionic interactions, and water-mediated interactions are shown by green, brown, and blue dotted lines. The phosphate backbones of certain nucleotides are shown as balls and sticks, whereas residues from SOX2 are shown as sticks. (**A**) Residues Lys57 to Tyr70 are involved in inter-residue interactions in the C-terminal helix of SOX2 and do not interact with DNA in the crystal structure. (**B**) During the MD simulations, the side chains of residues Lys57 and Arg60 from the SOX2 C-terminal helix form ionic interactions with DT8 and DT9. (**C**) Ionic interactions between SOX2 (residues Tyr72-Thr80) and DNA in the crystal structure are highlighted. Ile21 and Thr26 of OCT4 (shown as CPK) are known to form non-bonded interactions with Arg75 and Arg76 of SOX2. (**D**) Ionic interactions between SOX2 (Tyr72-Thr80) and DNA in a representative snapshot from the MD simulations. A transient ionic interaction is observed between Asp29 of OCT4 and Arg73 of SOX2. Residue numbering is based on the crystal structure of SOX2.

In the crystal structure of the SOX2/OCT4/FGF4-enhancer complex (PDB entry 1GT0), Arg75 and Arg76 form non-bonded interactions with Ile21 and Thr26 of OCT4 (Figure 8) [15]. During the MD simulations of the SOX2 and iPep systems, transient interactions were observed between Arg73 of SOX2 and the carboxylate group of Asp29 in OCT4 (Figure S7). SOX2 and OCT4 have complementary hydrophobic protein–protein interaction surfaces. However, it remains unclear if the iPep requires the presence of OCT4 for binding in the absence of structural determination data. Structural studies of SOX2 with various POU domains and analysis of genome-wide binding profiles in ESCs have shown that SOX2 interacts with its target DNA first [42], followed by the binding of the partner transcription factor proteins that have an intrinsic weak affinity for DNA [15,16,43]. The cooperative binding of SOX2 and its binding partners (i.e., POU members) increases the binding affinity of the ternary complex [44]. It has also been demonstrated that SOX2 and OCT3/4 interact directly through their DBDs in vitro in the absence of DNA, and assembly of the ternary complex by these two proteins in the presence of the FGF4-enhancer occurs cooperatively in embryonal carcinoma cells [45]. Furthermore, in cancer stem cells OCT proteins are also known to be co-expressed with SOX2 [46].

In order to determine the interface of the SOX2–OCT4 interaction, Remenyi et al. [15], introduced three mutations in its C-terminal region: R75E (FGF4-specific; m1), K57E, R60E (UTF1-specific; m2) and R60E, M64E (UTF1-specific; m3). None of these mutations were found to significantly impact the Sox2–DNA interaction, as the individual proteins bound to DNA in a similar manner to the respective wild-type protein. They also suggested a role for I21 and D29 of the POU domain of OCT4 in the protein–protein interaction with HMG in the POU/HMG/FGF4 complex and the POU/HMG/UTF1 complex. Our predictions of the free energy of interaction for the iPep mutant agree with the finding that the residues that form the helix (K57, R60 and M64) do not impact the interaction with DNA, whereas the C-terminal residues of SOX2/iPep are critical for interactions with DNA and OCT4.

The structure of the SOX2/OCT4/FGF4-enhancer complex suggests that the structuring of the C-terminus of the HMG domain of SOX2 is induced by the presence of the POU–HMG interface [15]. Residues Pro68–Lys79 are identical among all SOX members of the HMG family and heterodimer interface formation through the C-terminal HMG domain is a characteristic limited to the Sox subgroup of HMG proteins. Moreover, the complementary surface patch in OCT4 is also highly conserved among POU factors. The C-terminus of the HMG domain of SOX2, which is presumably unstructured in the absence of an interacting protein partner, is likely to be a major contributor to the formation of the ternary complex because the interaction of this portion of the protein with the minor groove of DNA increasing the HMG–DNA interface by about one-third of the total (420 Å^2^/1350 Å^2^) [15]. Recent solution NMR structural studies of SOX2 in the absence of OCT4 and the FGF4-enhancer (PDB entry 2LE4) have also revealed that its N-terminus is ordered while the C-terminus is disordered, suggesting that the C-terminal of SOX2 is likely to be unstructured in the absence of OCT4 and would likely exhibit diminished binding affinity to the FGF4-enhancer. MD simulations in the absence of OCT4 reveal the occurrence of a conformational change in the C-terminus of SOX2 (Figure S10) and increased flexibility in the FGF4-enhancer around the OCT4 recognition site. The free energy of binding (ΔG_binding_) of SOX2 predicted by the MM/GBSA method is −177.1 kcal/mol (Table S2), indicating a decrease in the affinity of SOX2 for the FGF4-enhancer in the absence of OCT4 and confirming the presence of cooperativity in the binding of SOX2 and OCT4 to the FGF4-enhancer in the ternary complex [44].

**Figure 9:**
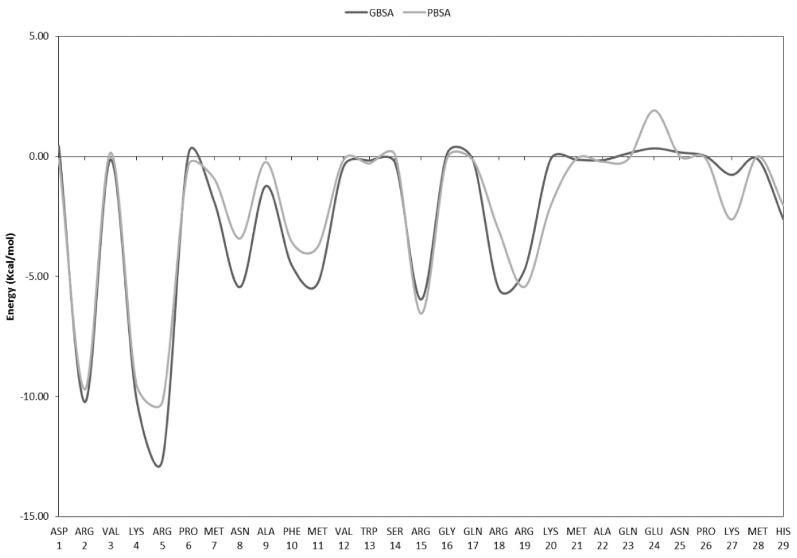
Free energy of binding decomposition per amino acid residue in the N-terminus of full-length SOX2. The free energies were calculated with both the PBSA and GBSA methods. Numbering is based on full-length SOX2.

A systems biology study identified short evolutionarily conserved recognition elements (CoREs) in the N-terminus of the HMG domain of SOX2 (Lys4-Ser14) that might serve as an anchor for DNA recognition, and that mutations in this region can disrupt its DNA binding potential and consequently its function [47]. Furthermore, a similar free energy decomposition analysis was also carried out on the N-terminus of the HMG domain of SOX2 (Figure 9). Three residues (Arg2, Lys4, and Arg5) are predicted to each have large, favorable contributions to the free energy of binding (≤ −10 kcal/mol), while six other residues (Asn8, Phe10, Met11, Arg15, Arg18, and Arg19) are predicted to each have significant favorable contributions (~−5–6 kcal/mol). Residues Ala9 to Ala22 form HMG helix-1. The side chains of three basic residues in this helix, Arg15, Arg18, and Arg19, form intermittent ionic interactions with the phosphates of DA45 and DA46, hydrogen bonds with the side chains of Asn30 and Ser34, and the phosphate of DG47, respectively. Recently, NMR and molecular docking studies highlighted the role of several amino acids (Val3, Lys4, Arg5, Met7, Arg15, and His29) in the N-terminus of the HMG domain of SOX2 in the interaction with Dawson-POM, which in turn inhibits the interaction of SOX2 with DNA [48], in agreement with the predictions above. Residues in the N-terminus of the HMG domain of SOX2 are also known to play a key role in DNA bending, thereby modulating transcriptional activity [49]. For example, the Asn8Gln mutation was reported to decrease DNA binding affinity and resulted in decreased DNA bending [49]. Consequently, a different iPep may be derived from the N-terminal conserved region of the HMG domain of SOX2, although an additional basic residue motif may be needed to act as a cell-penetrating sequence.

#### 2.6.2. Role of Water Molecules in the Interactions of SOX2 with DNA

As explained in Section 3, crystallographic water molecules found at the interacting interface of the SOX2/OCT4/FGF4-enhancer complex were explicitly retained as this maximized the integrity of the interactions observed in the crystal structure during the MD simulations. An analysis of H-bonds and water-mediated interactions in the MD simulations carried out with and without the inclusion of crystallographic water molecules, was done with reference to the hydrogen bonding interactions observed in the crystal structure (obtained from PDBsum), as shown in Table 2.

**Figure 10:**
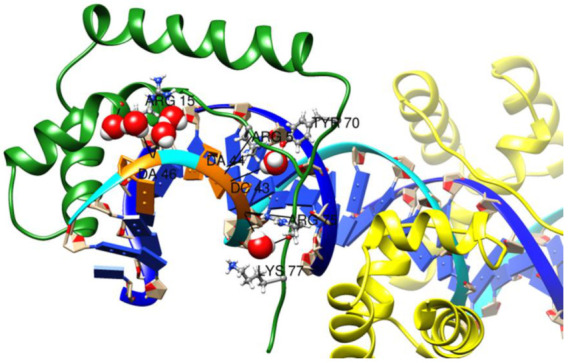
Snapshot taken from the simulation of the SOX2/FGF4/Oct4 complex. SOX2, FGF4, and OCT4 are shown with green, blue, and yellow ribbons, respectively. The interactions between Arg75-C43, Tyr70-DA44, and Arg15-DA46 are mediated by water molecules, which are shown as spheres. All water molecules within a 2.5 Å radius of the N- and C-termini of SOX2 were considered but are not shown for clarity. Hydrogen bonds are denoted by black lines.

In general, hydrogen bonds observed in the crystal structure were more likely to be retained in the MD simulation carried out with crystallographic water molecules. The loss of the hydrogen bond between DNA C3 and Ser31 in the simulation with crystallographic water molecules was replaced by a water-mediated hydrogen bond, which was observed during 8% of the simulation time. The low prevalence of the hydrogen bond between DNA T9 and Arg5 was found to be due to the large distance between the two residues (>4.0 Å in the crystal structure), suggesting that there is an intrinsic relatively low probability of formation of this weak hydrogen bond. While the prevalence of a water-mediated hydrogen bond between DNA C43 and Arg75 was found to be lower in the simulation with crystallographic water molecules, a new, direct hydrogen bond between DNA C43 and Arg75 (Figure 10) was observed during 55% of the simulation time.

The inclusion of crystallographic water molecules alongside the use of explicit water molecules in the MD simulations appears to be important for retaining the interactions between SOX2 and DNA observed in the crystal structure. It should be noted that previous studies have shown that the MM/PBSA method performs better when explicit water molecules mediating protein–protein/ligand interactions are retained [50,51].

## 3. Materials and Methods

### 3.1. Kaplan Meier Survival Curves

Kaplan Meier curves reflecting overall survival and progression-free survival in patients affected with breast cancer were obtained using datasets available at the cBioPortal (http://cbioportal.org/). The largest dataset in breast cancer (METABRIC) was chosen [52,53,54]. The analysis was performed with expression mRNA data from 1904 patients in total, 106 patients with low SOX2 mRNA levels, and 1798 patients with high SOX2 mRNA levels. Samples with a z-score >2 were considered as having high SOX2 expression and samples with a z-score <−2 were considered as low SOX2 expressors.

### 3.2. Identification and Quantification of SOX2 Peptides by MaxQuant

Individual LC-MS/MS raw files retrieved from the PRIDE database representing mass-spectrometry measurements of various human healthy tissues and cancer tissues, cell lines, and plasma samples were analyzed by MaxQuant version: 1.6.17.0(Max-Planck-Institute of Biochemistry, Germany). All the MaxQuant parameters were set as described previously by Wilhelm et al. [55]. Briefly, the MS/MS spectra were searched using the Andromeda search engine implemented in Maxquant against the custom-built merged FASTA database encompassing all the peptide sequences. MaxQuant analysis included an initial search with a precursor mass tolerance of 20 ppm, the results of which were used for mass recalibration. In the main Andromeda search, precursor mass and fragment mass had an initial mass tolerance of 6 ppm and 20 ppm, respectively. The search included variable modifications of methionine and oxidation, and N-terminal acetylation, and fixed modification of carbamidomethyl cysteine. The minimal peptide length was set to six amino acids and a maximum of two missed cleavages were allowed. The false discovery rate was set to 0.01 for peptide and protein identifications. For quantification of the peptides, the raw intensities were converted to ratios and then spectra for each peptide calculated, and the standard deviation of the logs of these ratios was fed for statistical analysis. The ratios reported represented by MaxQuant in biological replicates were used to calculate significant log2 changes.

### 3.3. Cell Line and Culture Conditions

The murine T11 cell line was kindly provided by C. Perou and L. Varticovski (University of North Carolina, Chapel Hill, USA, and National Cancer Institute, Bethesda, USA). This cell line recapitulates the claudin-low basal-like breast cancer subtype. MCF-7, HDEF, and PA-1 were purchased from ATCC. T11 cells were maintained in RPMI-1640 media supplemented with 10% fetal bovine serum (FBS) and 1% penicillin/streptomycin. MCF-7 cells were cultured in an MEM alpha medium containing 10% FBS, 1% sodium pyruvate, 1% sodium bicarbonate, 1% non-essential amino acids, and 1% antibiotic-antimycotic. The HDEF cells were cultured in a DMEM medium supplemented with 10% FBS and 1% antibiotic-antimycotic. PA-1 cells were cultured in a MEM alpha medium supplemented with 10% FBS and 1% antibiotic-antimycotic. All of the cells were cultured in a humidified 37 °C/5% CO2 incubator.

### 3.4. Cellular Internalisation Assay

To assess the cellular internalization of the SOX2 iPep fluorescence detection of the SOX2 iPep tagged with TAMRA was performed. T11 cells were seeded in coverslips and treated the day after for 2 h with the SOX2 iPep and with an alanine-mutated version of the SOX2 iPep at a concentration of 20 µM dissolved in a serum-free media. The iPep mutant has four amino acid substitutions with alanine: one lysine (Lys57) and one arginine (Arg60) in the nuclear localization sequence (NLS); one norleucine (Nle64) in the link between the NLS and the interference region, and two arginines (Arg75 and Arg76) in the interference region. After the treatments, cells were fixed with 4% formaldehyde, washed thrice, and nuclei were stained with Hoechst 33,258 at a final concentration of 1 ng/mL for 15 min. Next, coverslips were mounted in slides using SlowFade Diamond Antifade Mountant (ThermoFisher Scientific, VIC, Australia) and visualized using a Nikon Ti-E microscope. Images were taken using the 40× objective.

### 3.5. Cell Proliferation and Apoptosis Assay

Cell proliferation assays were performed in T11, MCF-7, HDEF, and PA-1 cells treated with vehicle control, 15 µM, 50 µM, and 100 µM of SOX2 iPep and iPep mutant for 0, 24, and 48 h. For T11 cells, 1500 cells were seeded per well in p96-clear plates and MTT reagent (Sigma-Aldrich, NSW, Australia) was added at the end of the treatments for one hour at a final concentration of 0.5 mg/mL. After one hour, MTT was aspired from the wells and 50 µL of DMSO was added. For MCF-7, HDEF, and PA-1 cells, 2000 cells were seeded per well in p96-white bottomed plates and processed by the CellTiter-Glo^®^ 2.0 luminescence assay protocol (Promega Corporation, NSW, Australia). Absorbance at 570nm was then read using a multiplate reader and time 0 of the vehicle control was considered to be 100% of cell proliferation. For the apoptosis assay, MCF-7 and PA-1 cells were seeded at 100,000 cells per well in p24-clear plates. The cells were treated with 100 µM of SOX2 iPep and iPep mutant for 24 h. Cell apoptosis was determined by TUNEL assay (In Situ Cell Death Detection Kit; Roche, VIC, Australia.

### 3.6. Western Blot

Cells were washed twice with PBS and lysed in a cold cell Lysis Buffer (Cell Signaling Technology, QLD, Australia) containing 1 mM phenylmethylsulfonyl fluoride (PMSF) then sonicated for 10 s at 10 mA. For each sample, 15 µg of protein was mixed with Laemmli sample loading buffer (Bio-Rad, NSW, Australia) supplemented with the reducing agent dithiothreitol (DTT), denatured at 95 °C for 5 min, loaded into Mini-PROTEAN precast gels (Bio-Rad, NSW, Australia), and subjected to electrophoresis at 100 V. The gel was then transferred to PVDF membranes (Bio-Rad, NSW, Australia) and blocked with 5% skim milk. The membrane was incubated overnight at 4 °C with the primary antibodies, anti-SOX2 (Cell Signaling Technology, QLD, Australia, #23064),and anti-tubulin (Sigma-Aldrich, NSW, Australia, #T1568). The signal was detected with Luminata Crescendo Western HRP Substrate (Millipore, NSW, Australia) using the ChemiDoc MP Imaging System (Bio-Rad, NSW, Australia).

### 3.7. Circular Dichroism (CD) Spectroscopy-

Peptides containing the cell-penetrating sequence can fold into distinct secondary structures, including α-helices and random coils compared to the full-length protein in the presence and absence of DNA. In the absence of NMR and X-ray data for the SOX2 iPep, structural characterization using circular dichroism (CD) was undertaken to estimate the extent of secondary structure content of the peptide. The sequence contained NorLeucine in a buffer. The SOX2-iPep was dissolved at a concentration of 50 µM in a phosphate buffer (50 mM) at a pH of 7.4. The samples were incubated for 4 h at 4 °C prior to the measurements. CD experiments were done using a Jasco J-815 spectropolarimeter with a path length of 1.0 mm in a rectangular Spectrosil quartz cuvette ((Starna, Baulkham Hills, Australia). All measurements were done in triplicate with standard sensitivity (100 mdeg), data pitch of 1 nm, a bandwidth of 1.00 nm, continuous scanning mode at a speed of 20 nm/min, and a response of 1.0 s. Absorbance was monitored over the range 260–190 nm. All experiments were preceded by blank measurements over three accumulated scans using the buffer only. The CD spectra obtained at each concentration were blank-subtracted. Secondary structure content was analyzed using a range of tools to ensure consistency. The analysis reported used the CDSSTR method with the reference set 7 [56], which was specifically optimized for the wavelength range used.

### 3.8. Molecular Dynamics Simulation of the Interaction of the SOX2 iPep with DNA

There are two three-dimensional structures available describing complexes of SOX2 with DNA and a partner transcription factor. PDB entry 1O4X is a solution NMR structure of the complex of the HMG domain of SOX2 with the POU domain of Oct1 and Hoxb1 DNA [16], while PDB entry 1GT0 is a crystal structure of the complex of the HMG domain of SOX2 with the POU domain of OCT4 and the FGF4-enhancer [15].

The structure in PDB entry 1GT0 was chosen as it contains a number of bound water molecules that may be important for the specific interactions of the proteins with DNA. In addition, this structure contains a 24-base pair long DNA sequence (FGF4-enhancer) with two intrastrand GpG sequences that may serve as potential cisplatin binding sites. The structure of OCT4 in 1GT0 was in fact generated by homology modeling based on the crystal structure of the ternary complex OCT1/SOX2/FGF4-enhancer, with 60% identity shared between the POU domains of OCT1 and OCT4.

Missing side-chain atoms in PDB entry 1GT0 were modeled using BIOVIA Dis-covery Studio (Dassault Systèmes, Vélizy-Villacoublay, France) Discovery Studio 4.0 (Biovia). The N- and C-termini of SOX2 and OCT4 were capped using acetyl (ACE) and N-methyl amide (NME) groups, respectively. Missing residues in the linking loop region Ser78-Glu96 between the POU and homeodomain in OCT4 were not modeled since this region, consisting of prolines, glycines, and serines was not observed in the electron density maps, indicating that it is disordered in the crystal, and does not appear to have any effect on the affinity of DNA [14]. Furthermore, it has been speculated that the linker is readily accessible to proteases both in solution and when bound to DNA during proteolysis of the POU domain. Therefore, the two OCT4 domains were also terminated with appropriate capping groups. Modeling and visualization were done with UCSF Chimera (Resource for biocomputing, visualization, and informatics, University of California, San Francisco, CA, USA) [57].

### 3.9. Molecular Dynamics Simulations

MD simulations of the complexes were carried out using the ff12SB force field in the Amber 12 package [58]. The complex was solvated in a triclinic periodic box of TIP3P water molecules [59] with a minimum of 10.0 Å between the edges of the protein–DNA complex and the simulation box. Preliminary simulations revealed that water molecules found in the crystal structure at the protein–DNA interface needed to be retained to account for interactions mediated by water and maintain the interactions between all macromolecules. A further 41,639 water molecules were added (35,845 water molecules were added to the complex containing the iPep) to fully hydrate the complex. Net charges in the protein–DNA complexes were neutralized by adding appropriate numbers of Na+ counter ions. The solvated protein–DNA complexes were first optimized by conducting 500 steps of steepest descents and 10,000 steps of conjugate gradients energy minimization while keeping all atoms of the complex restrained to their initial positions using a harmonic potential with a force constant of 100.0 kcal/mol/Å. A second minimization was carried out with a weak harmonic potential using a force constant of 10.0 kcal/mol/Å, followed by a third minimization with all restraints removed. Each system was then heated from 150 K to 300 K for 0.5 ns with a weak harmonic restraining potential (with a force constant of 2.0 kcal/mol/Å) applied to the protein–DNA complex in order to equilibrate the system without undesirable drift in the structure. A subsequent 0.5 ns MD simulation with a weak harmonic restraining potential (with a force constant of 2.0 kcal/mol/Å) on the protein–DNA complex was carried out at a constant pressure of 1 atm and a temperature of 300 K to equilibrate the system at the appropriate aqueous density. During the heating and equilibration of the iPep/OCT4/FGF4-enhancer complex, weak harmonic restraints (with force constants of 5.0 kcal/mol/Å^2^ during heating and 1 kcal/mol/Å^2^ during equilibration) were also applied to the phosphate (OP1) atom in DT9 of the DNA strand and one carbon (CD) and two nitrogen atoms (NE and NH1) in Arg 191 (Arg 246 in PDB structure 1GT0) in order to retain the corresponding electrostatic interactions and maintain the stability of the iPep–DNA complex. The iPep/OCT4/FGF4-enhancer complex was allowed full flexibility and all restraints were removed during the production stage of the simulations, which were run for 9.0 ns at constant pressure and temperature (NPT ensemble). Atomic coordinates were saved every 5 ps. The non-bonded cut-off applied was 8.0 Å and a continuum correction was used for the energy and pressure for vdW interactions beyond the cutoff. The particle mesh Ewald (PME) method was used to treat long-range electrostatic interactions under periodic boundary conditions. All bonds involving hydrogen atoms were constrained using the SHAKE algorithm. A time step of 2.0 fs was used in all MD simulations. In order to obtain accurate statistical averages and enhance the exploration of conformational phase space, ten runs of both the SOX2/OCT4/FGF4-enhancer and iPep/OCT4/FGF4-enhancer systems were conducted for 9.0 ns each with different initial random velocities satisfying a Maxwell distribution [60]. Hydrogen bonds and ionic interactions in the protein–DNA complexes were monitored throughout the simulations. UCSF Chimera was used for visualization of the simulation trajectories. Running averages of density, temperature, pressure, kinetic and potential energies of the systems were monitored during the simulations to ensure that proper equilibration had been achieved.

A similar protocol was followed to simulate the SOX2/FGF4-enhancer complex (without OCT4) in a triclinic period box with 21,019 TIP3P water molecules and an appropriate number of counter ions. Water molecules found at the protein–DNA interface in the crystal structure were also retained.

### 3.10. Free Energy of Binding Calculations

Calculations of the free energy of binding were performed with the MM/PBSA and MM/GBSA methods to characterize the various energy and entropy contributions to the protein–DNA interactions of these systems [61]. These methods have been employed previously to study the mutually exclusive cooperative binding of OCT4 with SOX2 and SOX17 to canonical (sequence related to CTTTGTCATGCAAAT) and compressed composite motifs (i.e., with one base pair missing between individual binding sites compared to the canonical motif) [62].

The MM/PBSA and MM/GBSA methods are used to predict free energies of binding by combining calculations of the molecular mechanics energy of interaction from MD simulations in an explicit solvent with calculations of the electrostatic contribution to the free energy of solvation using implicit solvation methods such as the Poisson-Boltzmann (PB) or the Generalized Born (GB) approaches, and the non-polar contribution to the free energy of solvation from calculations of the total surface area [63].

The free energy of binding (ΔG_binding_) can be defined as:

ΔG_binding_ = ΔG_gas_ + ΔG_sol-cmplx_ − [ΔG_sol-prot_ + ΔG_sol-lig_] (1)

ΔG_gas_ is the energy of interaction between protein and ligand in the gas phase, while ΔG_sol-cmplx_, ΔG_sol-prot_ and ΔG_sol-lig_ are the free energies of solvation of the protein–ligand complex, the protein, and the ligand, respectively.

Each ΔG component is the sum of the average molecular mechanics energy (ΔE_MM_), the free energy of solvation (ΔG_solv_), and molecular entropy (T_ΔS_):

ΔG = ΔE_MM_ + ΔG_solv_ − T_ΔS_ (2)

E_MM_ is the sum of the internal bond, angle and torsional energies (ΔE_bond_, ΔE_angle_, and ΔE_tor_), the non-bonded electrostatic energies (ΔE_elec_), and van der Waals energies (ΔE_vdw_):

E_MM_ = E_bond_ + E_angle_ + E_tor_ + E_vdw_ + E_elec_ (3)

The free energy of solvation (G_solv_) is calculated as a correction term accounting for the polarization induced by the presence of the solvent (i.e., water) as well as hydrophobic hydration. Consequently, G_solv_ contains a polar contribution, which can be accounted by PB or GB models, and a non-polar contribution, which is assumed to be proportional to the solvent-accessible surface area. In the MM/PBSA method the free energy of solvation (G_solvated-PB_) is calculated as the sum of the PB polar contribution (G_PB_), the non-polar energy (G_non-polar_), and the dispersion energy (E_dispersion_):

G_solvated-PB_ = G_PB_ + G_non-polar_ + E_dispersion_ (4)

A variant of the PBSA method was followed using Tan & Luo radii [64,65] for the protein. In this method, the non-polar solvation energy is split into two terms: the attractive (dispersion) and repulsive (cavity) interactions. E_dispersion_ is calculated by a numerical determination of the solvent-accessible surface area [66]. The non-polar term of the solvation free energy is calculated using the equation γSASA + b, where SASA is the solvent-accessible surface area, γ = 0.03780 and b = 0.5692 [66].

In the MM/GBSA method the free energy of solvation is calculated as the sum of the generalized GB contribution (G_GB_) and the surface energy (G_surface_):

G_solvated-GB_ = G_GB_ + G_surface_ (5)

In both implicit solvation methods, the free energy of solvation of the protein complex was calculated from representative structures taken from the MD simulation production run trajectories. The MM-PBSA.py.MPI [67] module of Amber 12 and AmberTools 14 were used to compute the components of the free energy of binding by analyzing all 1800 snapshots during each production run that had been taken at 5.0 ps time intervals. For the PBSA calculations a dielectric constant of 1 was used for the macromolecules and 80 for the aqueous solvent with an assumed ionic strength of 0.1 M. A modified GB model (igb = 5) [68] was used with constant values α = 1.0, β = 0.8 and γ = 4.85.

The individual amino acid contributions to the free energies of binding were calculated with both PBSA and GBSA approaches using the free energy decomposition [69,70] function in the MM-PBSA.py.MPI module with the parameter idecomp = 1, which denotes a per-residue decomposition with intramolecular 1–4 terms added to the internal potential energy terms. All the energy components including the self-energy term of GB and the nonpolar part of solvation free energy were decomposed during the analyses. These analyses did not take into account the internal strain energy.

All free energies were obtained as averages over the ten independent 10 ns MD simulations carried out for these systems. The single-trajectory approach was followed, in which the structures of each member of the macromolecular complex (e.g., SOX2 or iPep) in their unbound state were extracted from the simulations of the complex, rather than from independent simulations in an aqueous solution. Consequently, in this approach the assumption is made that each member of a macromolecular complex does not change conformation upon binding, resulting in the changes to the internal (bond, angle, and torsional) energies upon complexation being equal to zero.

### 3.11. Molecular Entropies

Molecular entropies consist of translational, rotational and vibrational contributions. The elastic network (EN) method was used to calculate vibrational entropies, as it has been reported that this approximation works well for the calculation of the vibrational entropy contribution to the free energy of binding of macromolecular complexes [71]. A variation of the RTB (rotations-translations of blocks) method with a potential cut-off of 8.0 Å), a force constant of 10.0 kcal/Å^2^/mol) and a block size of three was followed using the programmes GENENMM and DIAGRTB implemented in ΔΔPT [72]. The FREQ/EN programme was used to calculate vibrational frequencies, free energy, and entropy-based on the Schlitter approximation [73]. Fifty snapshots taken at 180 ps time intervals from each production run were used for these entropy calculations. The translational and rotational entropies were approximated analytically [74] with the ptraj program in Amber 12.

## 4. Conclusions

In this work, we show the effects of the SOX2-iPep in cancer cells and a negative trend between SOX2 mRNA levels and overall survival in breast cancer patients. Encouraged by the role of iPep, the interactions and predictions of the binding affinities of the HMG domain of SOX2 and a SOX2-iPep to FGF4-enhancer DNA in the presence of the partner transcription factor OCT4 were calculated using MD simulations. Specific binding of the C-terminus of SOX2 is associated with a sharp bending of the DNA molecule as well as positioning of partner proteins such as OCT4, mostly through a ring of hydrophobic interaction. Ionic interactions between Arg73 and Arg75 in SOX2 and Asp29 in OCT4 are the only sequence-specific interactions between the POU and HMG domains. Predictions of the free energy of binding suggest that the iPep can maintain a stable conformation and binding affinity to DNA. A free energy decomposition analysis per amino acid residue of the iPep compared to SOX2 revealed that the iPep is able to retain most of its binding affinity to DNA. Interestingly, the free energy of binding terms arising from the interactions and solvation of electric charges are more favorable in SOX2 than the corresponding iPep. This suggests that the presence of water molecules at the interacting interface of the HMG domain of SOX2 with DNA reduces the magnitude of the favorable electrostatic interactions upon binding, although this effect appears to have a relatively smaller impact in SOX2 compared to the iPep due to the presence of many other charges in the protein. An iPep derived from the N-terminal region of SOX2, which is also responsible for the bending of DNA, is also predicted to have a favorable total free energy of binding while lacking a protein–protein binding interface with OCT4. Free energy decomposition analysis also investigated the role of residues involved in the recognition of the FGF4-enhancer. Conserved regions Lys4-Ser14 and Pro68-Arg76 in the HMG domain of SOX2 are predicted to make major contributions to the affinity for DNA, mainly arising from the formation of ionic interactions between the phosphate backbone of DNA and basic amino acids in SOX2.

Earlier experimental studies of the FGF4-enhancer have established that interactions between SOX2 and OCT proteins are necessary for high-affinity binding and recognition of DNA. The resulting ternary complex is active in ESC and embryonal carcinoma cells. The C-terminal region of SOX2 is unstructured in the absence of OCT4 interactions and results in lower binding with DNA. Overall, these findings show that strong cooperation between TFs OCT4 and SOX2 (and a peptide derived from it) is important for binding to their specific enhancer sites and for the high levels of expression of OCT4/SOX2 in carcinomas and ESCs, with both factors providing great promise for therapeutic applications.

## Figures and Tables

**Table 1:** Predicted average free energies of binding of SOX2 and iPep to Figure 4. enhancer in the presence of OCT4. Energies are reported in kcal/mol.

Energy Term	MM/GBSA		MM/PBSA	
	SOX2	iPep	SOX2	iPep
ΔE_vdw_	−197.2	−84.5	−197.2	−85.5
ΔE_elec_	−8423.8	−4552.3	−8423.8	−4552.3
ΔG_gas_ (ΔE_vdw_ + ΔE_elec_)	−8620.9	−4636.9	−8620.9	−4636.9
ΔG_GB_	8407.9	4555.1		
ΔG_PB_			8376.2	4532.0
ΔG_surface_	−23.9	−11.3		
ΔG_non-polar_			−127.7	−60.7
ΔE_dispersion_			254.0	117.0
ΔE_elec_ + ΔG_GB_/ΔG_PB_	−15.9	2.8	−47.6	−20.4
ΔG_solvated_	8383.9	4543.8	8502.5	4588.2
ΔG	−237.0	−93.1	−118.4	−48.7
TΔS	−44.3	−37.3	−44.3	−37.3
ΔG_binding_	−192.7	−55.8	−74.1	−11.4

**Table 2:** Prevalence (% time) of hydrogen bonds and water-mediated interactions observed in the 1GT0 crystal structure during the MD simulations.

Hydrogen Bonds with Sox2	H_2_O Mediated Hydrogen Bond	Without Crystallographic Water Molecules	With Crystallographic Water Molecules
DC3-Ser31	No	7.5%	0%
DT4-Ser34	No	82.8%	99.1%
DG7-Trp41	No	53.9%	58.3%
DT9-Arg5	No	1.8%	2.0%
DG11-Arg75	No	15.6%	13.9%
DA42-Tyr72	No	67.3%	88.5%
DC43-Arg5	No	85.4%	88.2–90.0% *
DC43-Arg75	Yes	69.8%	30.3%
DA44-Val3	Yes	16.9%	18.5%
DA44-Tyr70	Yes	12.8%	8.7%
DA44-Val 3-Tyr70	Yes	61.9%	64.4%
DA46-Arg15	No	43.7–47.5%	65.0%
DG47-Asn30	No	48.6%	65.4%

* The larger value arises from the occasional replacement of the crystallographic water molecule with solvent molecules during the simulation.

## Data Availability

Not applicable.

## Supplementary Materials

The following are available online at https://www.mdpi.com/article/10.3390/ijms22179354/s1.
