# Supplementary material for: Design and Characterization of a Cell-Penetrating Peptide Derived from the SOX2 Transcription Factor
Source: Int J Mol Sci. 2021 Aug 28;22(17):9354. doi: 10.3390/ijms22179354 (PMC8431565; doi:10.3390/ijms22179354)
Supplement: Supplementary file 1 [file ijms-22-09354-s001.zip › ijms-1177507-supplementary/supporting/ijms-1177507-supplementary-final.pdf]

Design and Characterization of a Cell-Penetrating Peptide Derived from the SOX2 Transcription Factor

Neha S. Gandhi [1,2], Edina Wang [3], Anabel Sorolla [3,7], Yu Jie Kan [1], Adil Malik [5,6], Jyotsna Batra [5,6], Kimberly A. Young [1], Wan Jun Tie [3], Pilar Blancafort [3,4,*] and Ricardo L. Mancera [1,*]

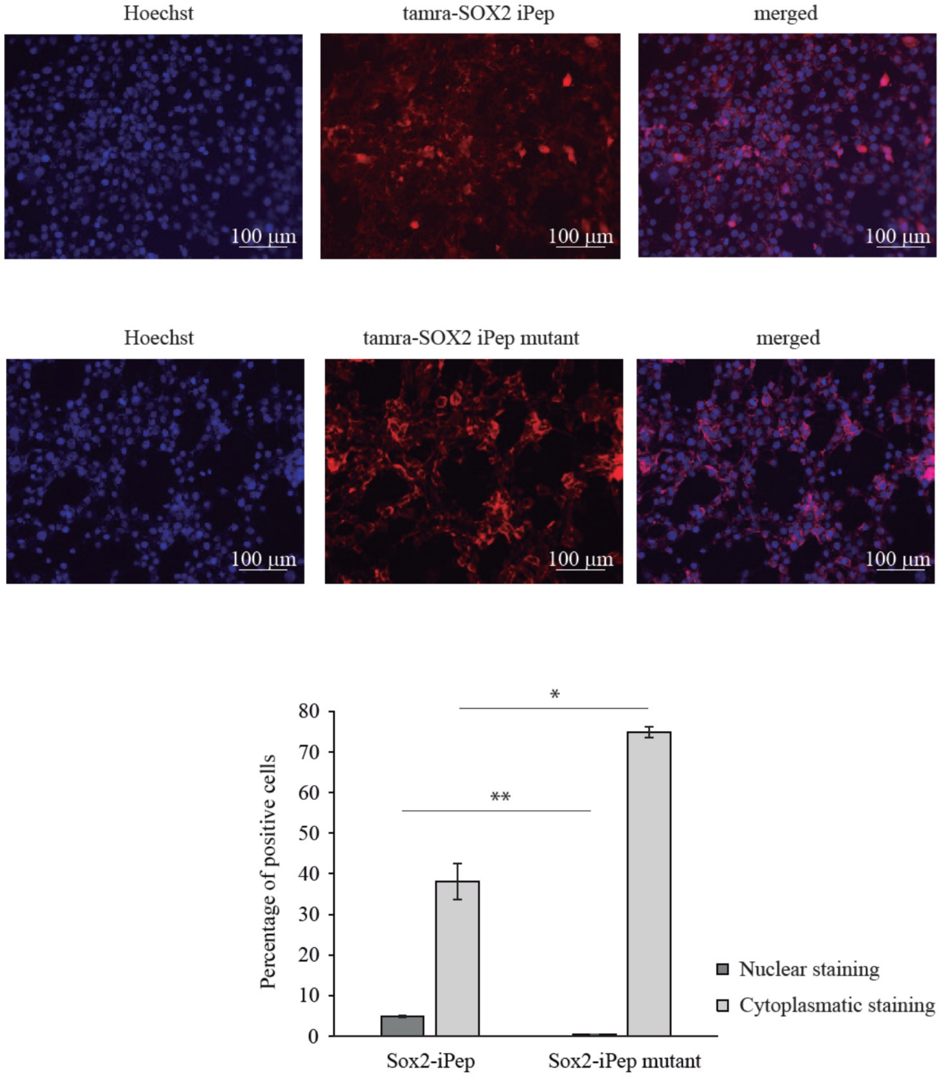

Figure S1 Representative fluorescence images of T11 cells treated for two hours with tamra-Sox2 iPep (upper panel) and with tamra-Sox2 iPep mutant (lower panel). Hoechst images, tamra images and merged images are shown for the two iPeps. Graph representing the percentage of cells with nuclear staining and cytoplasmatic staining is shown at the bottom. Treatment conditions were compared statistically using a two-tailed unpaired t-test. Scalebars indicate 100 µm. Data are represented as mean ± SEM. ** means $p<0.005$ and *** means $p<0.0005$.

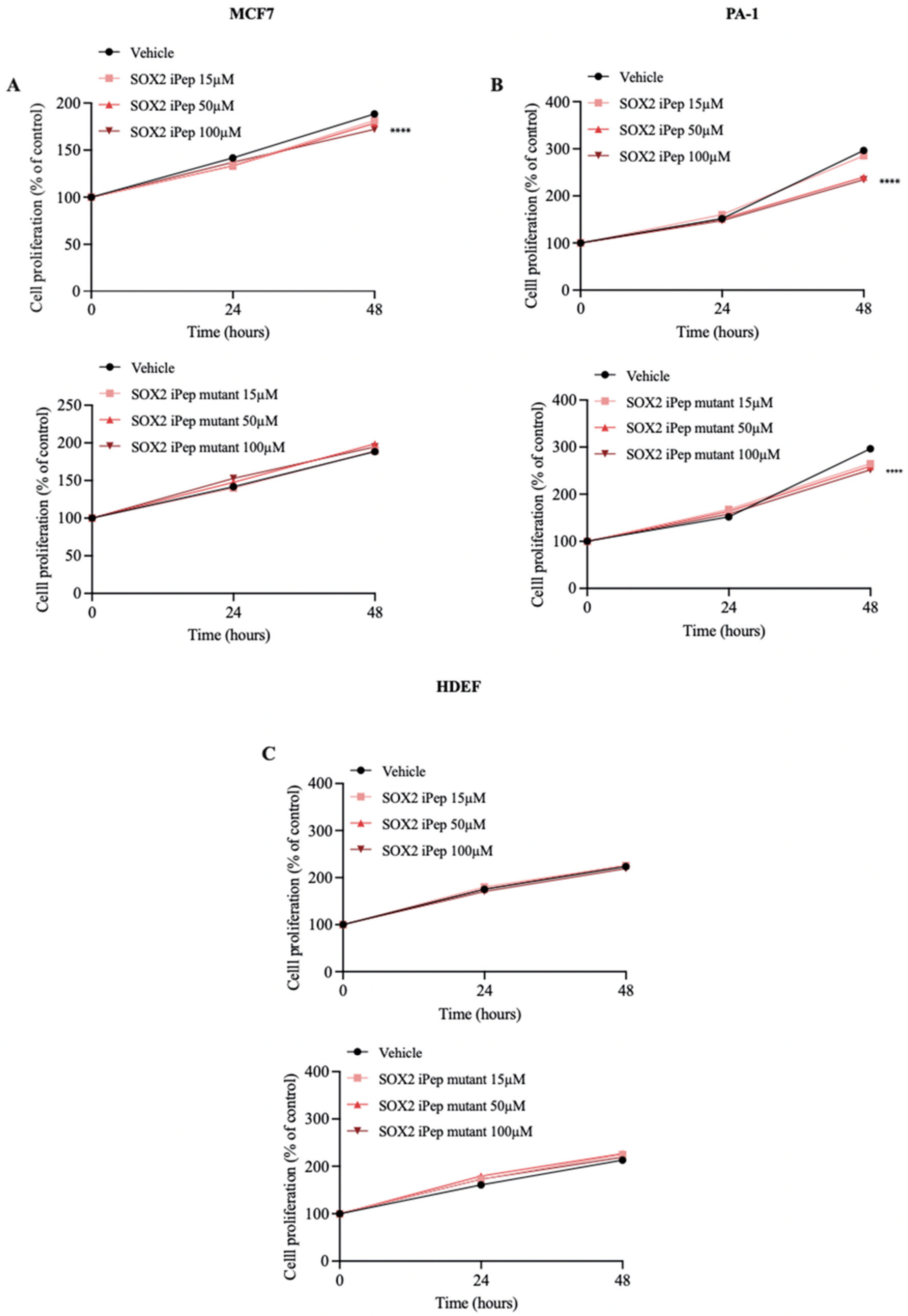

Figure S2 Graphs of the proliferation assays in (A) MCF7, (B) PA-1 cells (C) HDEF when treated with 15, 50 and 100 µM of SOX2-iPep (upper panel) and SOX2-iPep mutant (lower panel) for 0, 24 and 48 hours. Treatment conditions were compared statistically to the vehicle control using a multiple unpaired t-test. Data are represented as mean ± SEM. **** means $p<0.0001$.

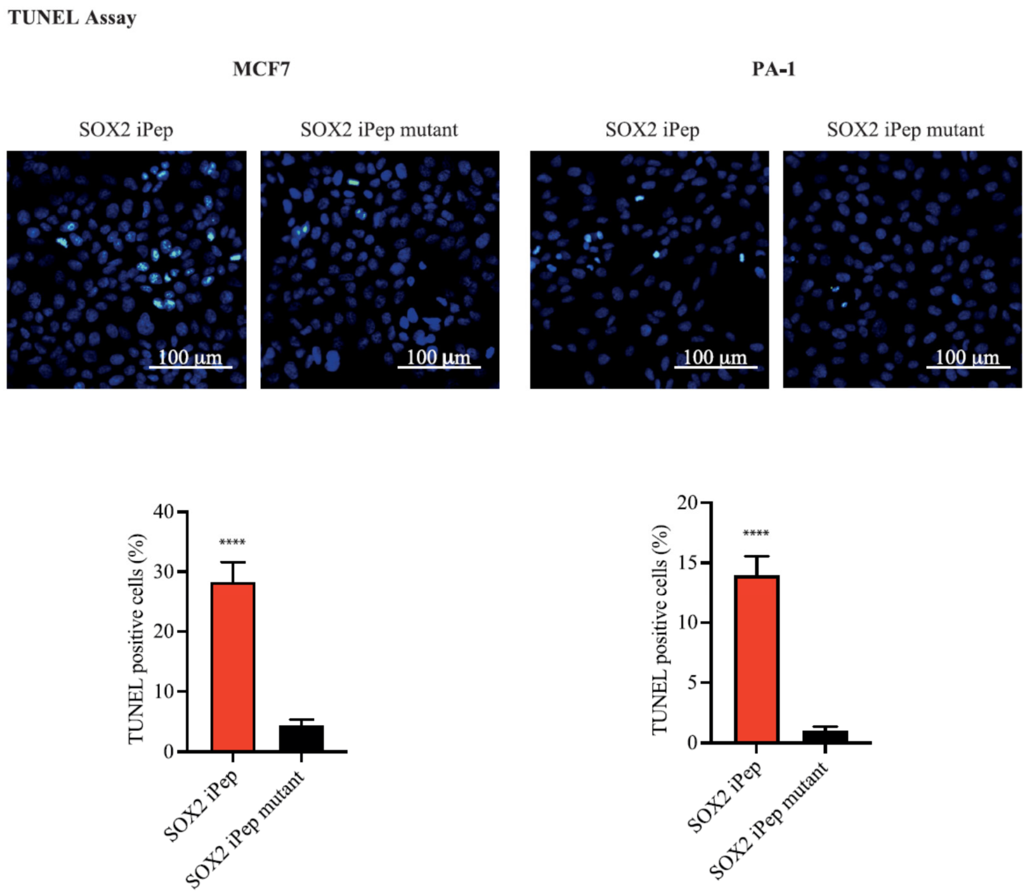

Figure S3 Representative images of MCF7 cells and PA-1 cells treated with SOX2-iPep and SOX2-iPep mutant at 100 µM for 24 hours and stained with TUNEL and Hoechst (upper panel). Graphs showing the percentage of TUNEL positive cells corresponding to the quantification of the images (lower panel).

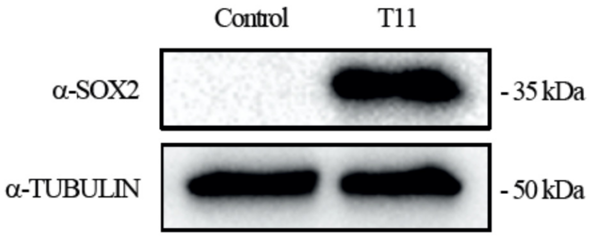

Figure S4 Western Blot in T11 cells and control cells (NIH/3T3) for the detection of SOX2. Tubulin was used as loading control.

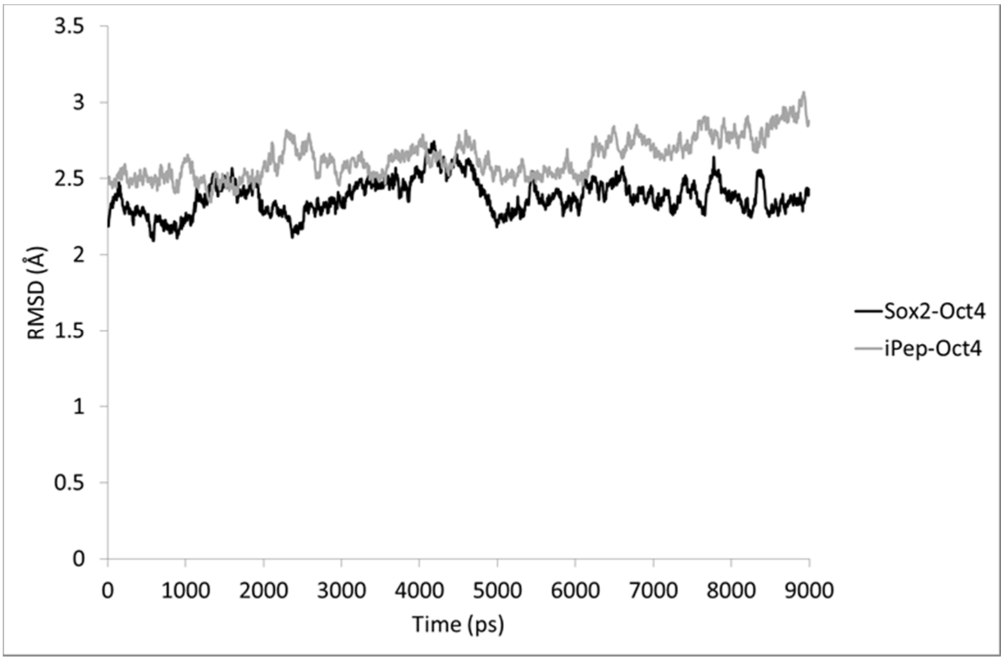

Figure S5. Time evolution of the RMSD of the coordinates of the protein main chain (Cα, C and N) of Sox2/Oct4 and iPep/Oct4 with respect to the initial coordinates in the crystal structure in the SOX2/Oct4/FGF4-enhancer complex.

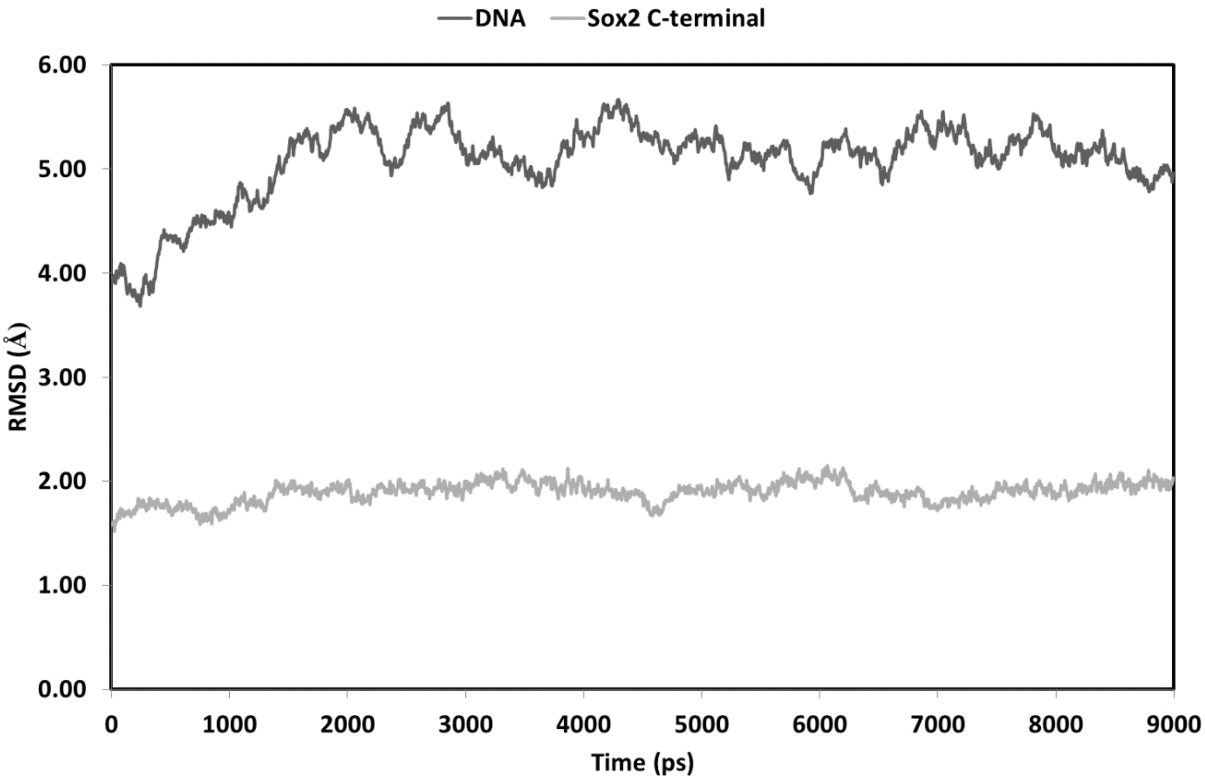

Figure S6. Time evolution of the RMSD of the coordinates of the protein main chain (Cα, C and N) of the C-terminus of Sox2 and the FGF4-enhancer backbone with respect to the initial coordinates in the crystal structure from a simulation of the Sox2/FGF4-enhancer complex in the absence of Oct4.

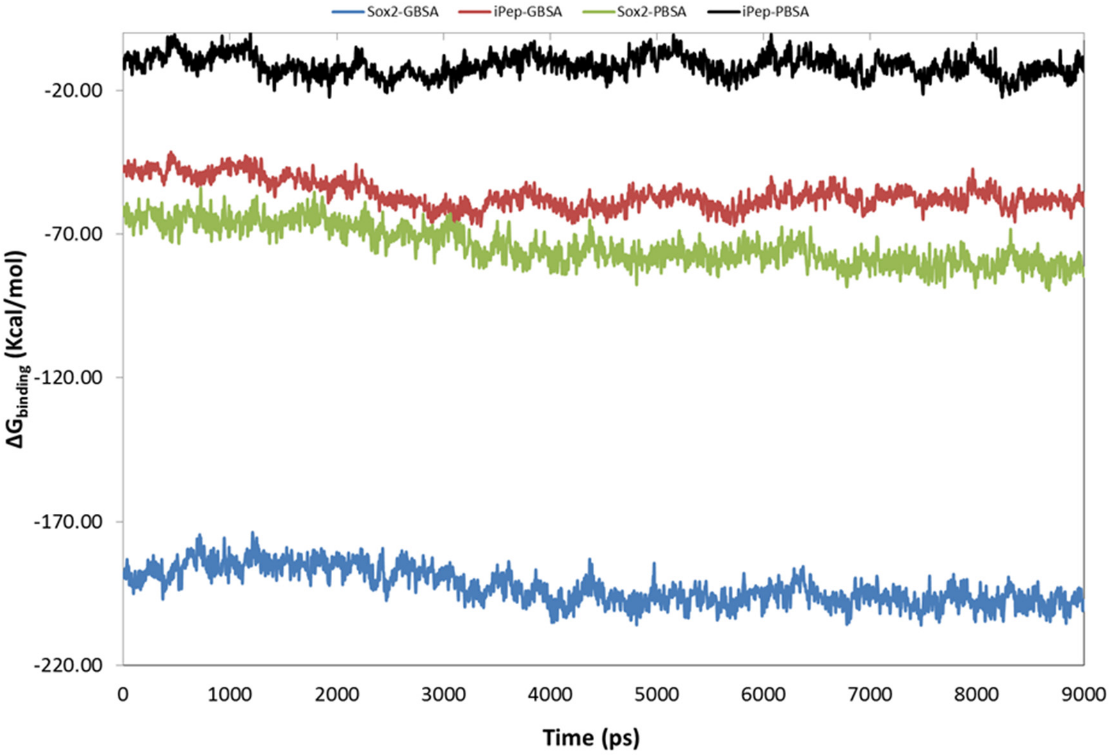

Figure S7. Time evolution of the average ΔG of binding of SOX2/OCT4/FGF4-enhancer and iPep/OCT4/FGF4-enhancer systems calculated with the MM/GBSA and MM/PBSA methods over ten separate simulations.

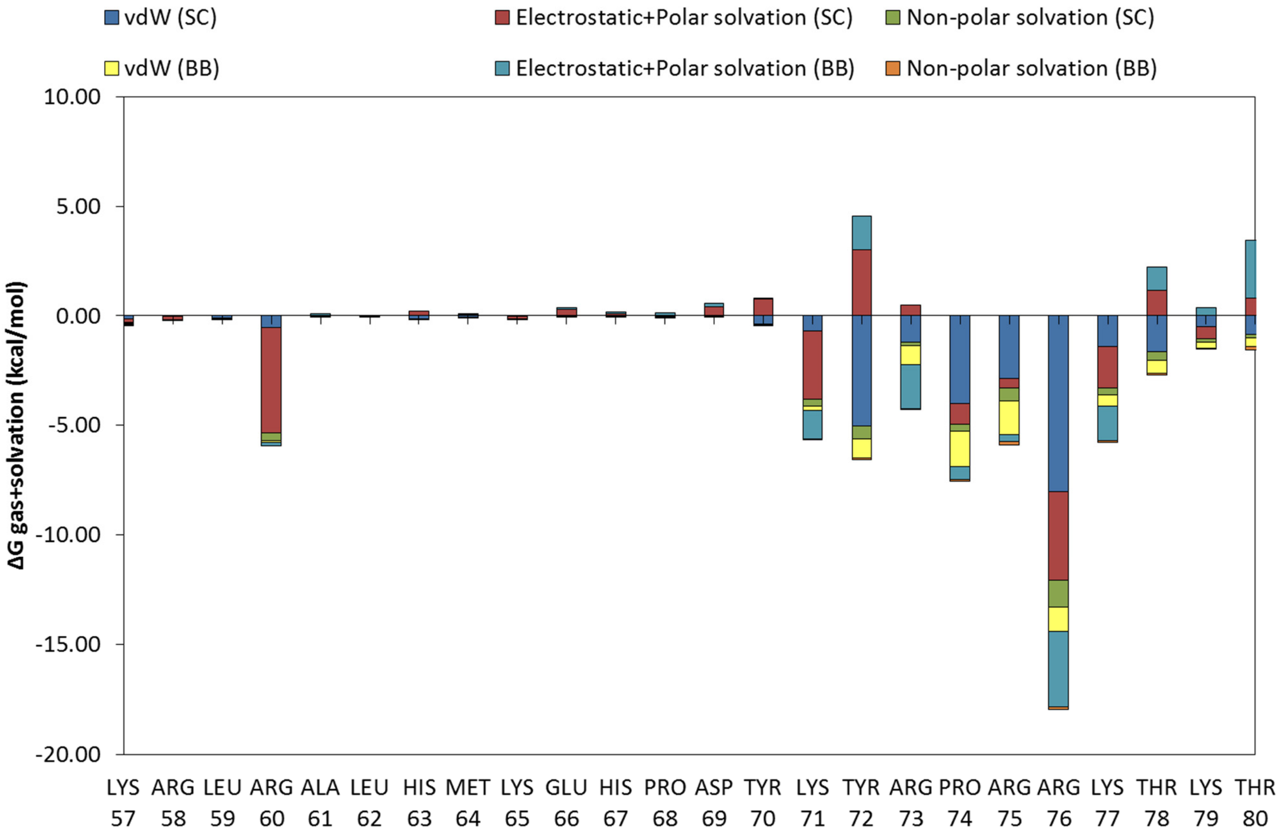

Figure S8. Decomposition of $\Delta G_{gas+solv}$ on a per-residue basis into contributions from van der Waals interactions (vdW), the sum of electrostatic interactions and polar solvation free energy, and the non-polar term of the solvation free energy for the C-terminal residues of the HMG domain of Sox2. The free energy components were calculated using the GBSA method and are shown separately for backbone (BB) and sidechain (SC).

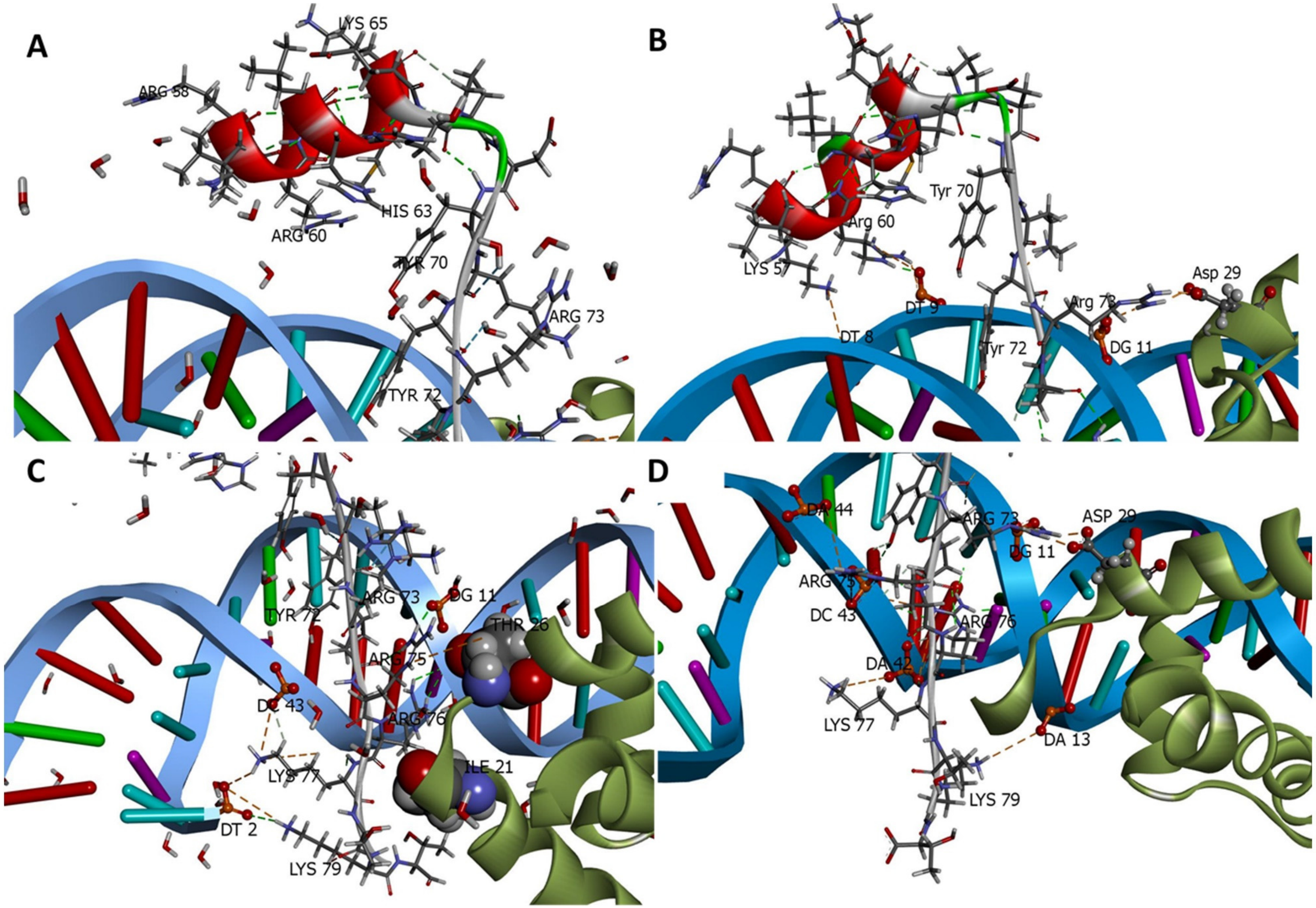

Figure S9. Interactions of residues in SOX2 (chain E) corresponding to the iPep in PDB entry 1GT0. The SOX2 region corresponding to the iPep is shown as ribbon and coloured according to secondary structure. The FGF4-enhancer (DNA) and OCT4 are shown in blue and green ribbon representation, respectively. Hydrogen bonds, ionic interactions and water-mediated interactions are shown by green, brown and blue dotted lines. The phosphate backbone of certain nucleotides are shown as ball and sticks, whereas residues from SOX2 are shown as sticks. A) Residues Lys57 to Tyr70 are involved in inter-residue interactions in the C-terminal helix of SOX2 and do not interact with DNA in the crystal structure. B) During the MD simulations, the side chains of residues Lys57 and Arg60 from the SOX2 C-terminal helix form ionic interactions with DT8 and DT9. C) Ionic interactions between SOX2 (residues Tyr72 - Thr80) and DNA in the crystal structure are highlighted. Ile21 and Thr26 of OCT4 (shown as CPK) are known to form non-bonded interactions with Arg75 and Arg76 of SOX2. D) Ionic interactions between SOX2 (Tyr72 - Thr80) and DNA in a representative snapshot from the MD simulations. A transient ionic interaction is observed between Asp29 of OCT4 and Arg73 of SOX2. Residue numbering is based on the crystal structure of SOX2.

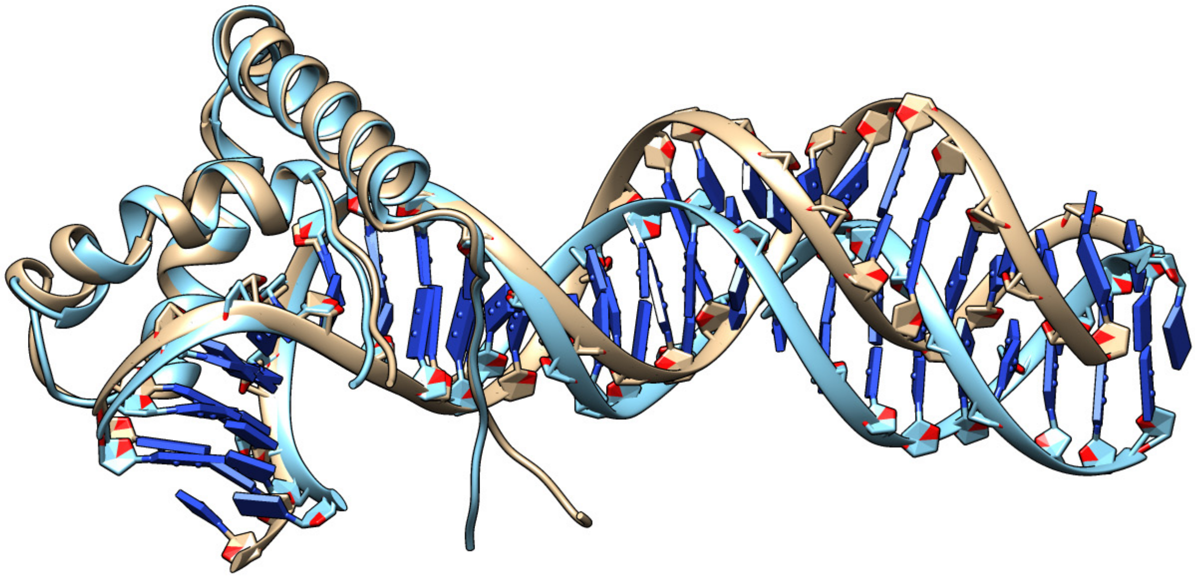

Figure S10. Superimposition of the Sox2/FGF4-enhancer complex (PDB entry 1GT0; Oct4 not shown for clarity) in blue onto a representative snapshot (in golden ribbon representation) taken from the simulation of the Sox2/FGF4-enhancer complex (in the absence of Oct4). The absence of Oct4 in the simulation affects the bending of Oct4 binding domain in the FGF4-enhancer, whereas the flexible C-terminal region in Sox2 moves closer to this Oct4 binding site.

Table S1. Interactions (hydrogen bonds and ionic interactions) of amino acid residues that contribute ≤ −5 kcal/mol to the free energy of binding in Sox2 from residue decomposition analysis. *SC =side-chain; BB=Backbone amide.

	Sox2		iPep	
Sox2 residue	Interacting residue	Fraction of frames that the interaction is present (corresponding donor in Sox2 residue)	Interacting residue	Fraction of frames that the interaction is present (corresponding donor in Sox2 residue)
Arg58 (SC)	Glu55 COO^- (Sox2)	0.08 (NE)<br>0.68 (NH1)<br>0.65 (NH2)	DT8 phosphate<br><br>DT9 phosphate<br><br>DA44phosphate<br><br>DA45 phosphate	0.4 (NE)<br>0.4 (NH2)<br>0.15 (NH1)<br>0.08 (NH2)<br>0.27 (NE)<br>0.31 (NH2)<br>0.23 (NE)<br>0.12 (NH1)
Arg60 (SC)	DT9 phosphate	0.64 (NE)<br>0.63 (NH2)	DT9 phosphate<br>DA45 phosphate	0.3 (NH1)<br>0.08 (NH1)<br>0.12 (NH2)
Lys71 (SC)	DT10 phosphate	0.8 (NZ)	DT10 phosphate	0.7 (NZ)
Tyr72 (SC)	DA43 N3 (base)	0.9 (OH)	DA43 N3 (base)	0.8 (OH)
Arg73 (SC)	DG11 phosphate<br><br>Asp29 COO- (Oct4)	0.51 (HE)<br>0.19 (NH2)<br>0.08 (NE)<br>0.56 (NH1)<br>0.46 (NH2)	DG 11 phosphate<br><br>Asp29 COO- (Oct4)	0.73 (NE)<br>0.69 (NH2)<br>0.4 (NH1)<br>0.70 (NH2)
Arg73 (BB)	DC43 phosphate	0.3 (NH)	DC43 phosphate	0.3 (NH)
Arg75 (BB)	DC43-phosphate	0.4 (NH)	-	
Arg75 (SC)	DC43-phosphate	0.62 (NE)<br>0.38 (NH2)	DC43 phosphate	0.7 (NH2)
Arg76 (SC)	DA41 N3 (base)	0.53 (NH1)<br>0.06 (NH2)	DA41 N3(base)<br>DT10 O2 (base)	0.4 (NH1)<br>0.7 (NH2)

	DT10 O2 (base)<br>DG11 N3 (base)<br>DG11 O4’ (ribose)	0.42 (NH2)<br>0.12 (NH2)<br>0.08 (NH2)	DG11 N3 (base)<br>DA42 O4’(ribose)	0.15 (NH2)<br>0.2 (NH1)
Lys77 (BB)	DA42 phosphate	0.5 (NH)	DA42 phosphate	0.7 (NH)
Lys77 (SC)	DA42 phosphate	0.2 (NZ)	DA43 phosphate	0.3 (NZ)
Lys79 (SC)	DA41phosphate<br>DA13 phosphate	0.3 (NZ)<br>0.1 (NZ)	-	

Table S2. Predicted average free energies of binding of Sox2 to the FGF4-enhancer in the absence of Oct4. Energies are reported in kcal/mol.

Energy term	MM/GBSA	MM/PBSA
ΔE_{vdw}	-179.9	-179.9
ΔE_{elec}	-9394.2	-9394.2
ΔG_{gas} (ΔE_{vdw}+ ΔE_{elec})	-9574.2	-9574.2
ΔG_{GB}	9378.1	
ΔG_{PB}		9329.3
$\Delta G_{surface}$	-21.6	
$\Delta G_{non\text{-}polar}$		-114.4
$\Delta E_{dispersion}$		228.6
ΔE_{elec}+ ΔG_{GB}/ ΔG_{PB}	-16.1	-64.9
$G_{solvated}$	9356.6	9443.6
ΔG	-217.6	-130.6
$T\Delta S$	-40.5	-40.5
$\Delta G_{binding}$	-177.1	-90.1
